# Spatiotemporal and temperature-dependent disconnect between ammonia oxidation and dark DIC fixation in deep oligotrophic Lake Constance

**DOI:** 10.1093/ismeco/ycaf201

**Published:** 2025-11-10

**Authors:** Jade Bosviel, Katharina Kitzinger, Francesca Vulcano, Franziska Klotz, Anton Legin, Petra Büsing, Thorsten Rennebarth, Joerdis Stuehrenberg, Hannah Marchant, Michael Wagner, Martin Wessels, David Schleheck, Marcel M M Kuypers, Michael Pester

**Affiliations:** Leibniz Institute DSMZ – German Collection of Microorganisms and Cell Cultures, Inhoffenstr. 7B, Braunschweig D-38124, Germany; Max Planck Institute for Marine Microbiology, Celsiusstrasse 1, Bremen D-28359, Germany; Centre for Microbiology and Environmental Systems Science, University of Vienna. Djerassiplatz 1, Vienna 1030, Austria; Leibniz Institute DSMZ – German Collection of Microorganisms and Cell Cultures, Inhoffenstr. 7B, Braunschweig D-38124, Germany; Department of Biology, University of Konstanz, Limnological Institute, Universitätsstrasse 10, Konstanz D-78457, Germany; Centre for Microbiology and Environmental Systems Science, University of Vienna. Djerassiplatz 1, Vienna 1030, Austria; Leibniz Institute DSMZ – German Collection of Microorganisms and Cell Cultures, Inhoffenstr. 7B, Braunschweig D-38124, Germany; Institute for Lake Research, LUBW State Institute for Environment Baden-Württemberg, Argenweg 50/1, Langenargen 88085, Germany; Max Planck Institute for Marine Microbiology, Celsiusstrasse 1, Bremen D-28359, Germany; Max Planck Institute for Marine Microbiology, Celsiusstrasse 1, Bremen D-28359, Germany; MARUM – Centre for Marine Environmental Sciences, University of Bremen, Leobener Str. 8, Bremen D-28359, Germany; Centre for Microbiology and Environmental Systems Science, University of Vienna. Djerassiplatz 1, Vienna 1030, Austria; Department of Chemistry and Bioscience, Aalborg University, Fredrik Bajers Vej 7H, Aalborg 9220, Denmark; Institute for Lake Research, LUBW State Institute for Environment Baden-Württemberg, Argenweg 50/1, Langenargen 88085, Germany; Department of Biology, University of Konstanz, Limnological Institute, Universitätsstrasse 10, Konstanz D-78457, Germany; Max Planck Institute for Marine Microbiology, Celsiusstrasse 1, Bremen D-28359, Germany; Leibniz Institute DSMZ – German Collection of Microorganisms and Cell Cultures, Inhoffenstr. 7B, Braunschweig D-38124, Germany; Chair of Microbial Physiology, Technical University of Munich, Emil-Ramann-Strasse 4, Freising D-85354, Germany

**Keywords:** nitrification, CO_2_ fixation, ammonia assimilation, chemolithoautotrophy, ammonia oxidizing archaea, hypolimnion, lake Constance

## Abstract

Deep oligotrophic lakes hold over 80% of global lake water. In their hypolimnion, ammonia oxidation (the first step of nitrification) and non-photosynthetic fixation of dissolved inorganic carbon (DIC) are key processes, presumably linked by large populations of ammonia-oxidizing archaea (AOA). We used stable isotope-based activity measurements to follow both processes below the thermocline and in the central hypolimnion in deep oligotrophic Lake Constance. Throughout seasons, they varied substantially below the thermocline peaking at 139.0 NH_4_^+^ nmol l^−1^ d^−1^ oxidized and 14.6 nmol DIC l^−1^ d^−1^ fixed. At the center of the hypolimnion, they were rather stable averaging 7.5 nmol NH_4_^+^ l^−1^ d^−1^ and 1.3 nmol DIC l^−1^ d^−1^, respectively. However, both processes did not correlate in their spatiotemporal and temperature-related dynamics. Temperature manipulations (5–20°C) confirmed this disconnect with ammonia oxidation peaking at 10°C while dark DIC fixation increased exponentially with temperature. DIC fixation of single AOA cells centered at 2.17 × 10^−18^ mol C cell^−1^ d^−1^, explaining only 11% of overall DIC fixation. Metatranscriptomic analyses supported this, revealing that most DIC-fixation pathway transcripts originated from RubisCO-encoding cryptophytes, cyanobacteria, and Alpha- and Betaproteobacteria, rather than AOA or other nitrifiers. These non-nitrifier groups likely activated the Calvin cycle to maintain redox balance in the dark. Our findings provide a new perspective on nitrification-driven chemolithoautotrophy in oligotrophic lake hypolimnia, with freshwater AOA contributing a minor part to dark DIC fixation, likely explaining decoupled dynamics of ammonia oxidation and dark DIC fixation.

## Introduction

Climate change caused average lake temperatures to increase globally by 0.34°C per decade since 1985 [1], with projections of an additional 1–4°C by 2100 [2, 3]. Consequences include shorter ice cover, earlier and prolonged thermal stratification, and warmer summer water temperatures [1], all affecting lake biogeochemistry and productivity [4–6]. While photosynthesis-driven primary productivity has been frequently in the focus of global change research in lakes, conversion of dissolved inorganic carbon (DIC) to organic matter in the dark, e.g. by chemolithoautotrophic microorganisms such as nitrifiers, received less attention [7–10].

Most of the world’s largest lakes are deep oligotrophic lakes. Together they comprise more than 80% of lake water by volume globally [11]. In aphotic and fully oxygenated waters of such lakes, a major part of DIC fixation pathway-encoding genes is related to nitrifiers [12, 13], i.e. chemolithoautotrophic microorganisms converting ammonia via nitrite to nitrate. Ammonia oxidation to nitrite is the first and typically the rate limiting step in nitrification [14], catalyzed either by ammonia oxidizing archaea (AOA) [15, 16] or bacteria (AOB) [17]. Both interact with nitrite oxidizing bacteria (NOB) [18], which oxidize nitrite further to nitrate. Alternatively, comammox bacteria can oxidize ammonia directly to nitrate [19, 20]. AOA (phylum *Nitrososphaerota*) typically dominate in deep oligotrophic lakes [21], comprising up to 39% of prokaryotic picoplankton in the hypolimnion [13, 22–27]. The Great Lakes may represent an exception with a predominance of AOB [28]. Typically, AOA are associated with NOB of the genus *Nitrospira* in these waters [13, 28–31]. While freshwater nitrifier composition has been studied, their contribution to dark DIC fixation remains underexplored [10, 29]. Even more so, the *in situ* temperature dependency of freshwater nitrification and dark DIC fixation is not understood at all.

Among the seven known DIC fixation pathways [32–34], the 3-hydroxypropionate/4-hydroxybutyrate (HP/HB), the reductive citric acid (rTCA), and the Calvin-Benson-Bassham (CBB) cycles dominate in oxygenated, aphotic pelagic zones [9, 12, 13, 35]. The HP/HB cycle is restricted to archaea, including an energy-efficient variant in AOA, and depends on the key enzymes 4-hydroxybutyryl-CoA dehydratase and acetyl-CoA/propionyl-CoA carboxylase [32, 36]. Genes encoding the former (*hcd*) or the beta subunit of the latter (*accB*) have been used to identify respective archaea in the environment [9, 12, 13, 37–40]. The rTCA cycle operates in phylogenetically and metabolically diverse bacteria [32, 41], including nitrite-oxidizing *Nitrospira* and their comammox subclades [18]. It typically relies on the ATP-dependent citrate lyase, with its alpha subunit-encoding *aclA* often targeted in environmental studies [9, 12, 13, 35]. The CBB cycle, widespread across all domains of life, occurs in many phototrophs but also chemolithotrophs [32, 42–44] such as all known AOB [45] and several NOB genera [18]. It depends on ribulose-1,5-bisphosphate carboxylase/oxygenase (RubisCO), particularly its form I and II variants [43, 44]. The large subunit of RubisCO is encoded by *cbbL*, a widely used marker gene for CBB-utilizing autotrophs [9, 12, 13, 35]. Importantly, the CBB cycle can also support intracellular redox balancing in photoheterotrophs and has been suggested to function in the same ways in strict organoheterotrophs [32, 46]. Besides these dedicated DIC fixation pathways, DIC can enter biomass through carboxylation steps during anaplerotic reactions, biosynthesis, or heterotrophic metabolism [47].

Lake Constance is ideal to study the link between dark DIC fixation and ammonia oxidation. This deep, oligotrophic lake is well characterized [48] and displays a fully oxygenated water column down to the bottom [48]. The nitrifier community is mainly found in the hypolimnion [30] and dominated by *Candidatus* Nitrosopumiulus limneticus [26], a widespread AOA in Eurasian lakes [21]. Its relative abundance (8–39%) [26, 30] is strongly correlated to nitrite-oxidizing *Nitrospira* species [26], which are typically one order of magnitude less abundant (0.3–1.1%) [30]. AOB affiliated with *Nitrosomonadaceae* occur as minor populations in the hypolimnion (0.02%–0.23%) [30], but can bloom up to 1.9% in the metalimnion [30]. Comammox bacteria are typically not detected by PCR-based assays [30], though metagenomics has revealed their presence at very low abundance [26]. Interestingly, this resembles very well nitrifier population structures in the ocean with the exception that nitrite-oxidizing *Nitrospira* are replaced by *Nitrospina* [49]. Here, we studied spatiotemporal dynamics of ammonia oxidation and dark DIC fixation in Lake Constance across temperature gradients in the hypolimnion, using bulk process measurements, single-cell analyses, and metatranscriptomics with a focus on the dominating populations of ammonia-oxidizing archaea (AOA).

## Materials and methods

### Sampling

Lake Constance is a temperate, oligotrophic peri-alpine lake with an extensive hypolimnion reaching down to a maximum depth of 251 m [48]. The lake is warm-monomictic and characterized by a fully oxygenated hypolimnion throughout the year and total phosphorous concentrations averaging 6.2 μg l^−1^ [50, 51]. Samples were collected in northwestern Upper Lake Constance (LTER station Wallhausen; 47.75788° N, 9.12617° E) on board the R/V Lauterborn on the 17.03.2021, 19.05.2021, 14.07.2021, 08.09.2021, 17.11.2021, 13.01.2022, 24.03.2022, 15.06.2022, 08.09.2022, and 22.02.2024 (dd.mm.yyyy, Supplementary Fig. 1). No permits were required for site access or water collection.

### Environmental parameters and single cell quantification

Vertical profiles of temperature and chlorophyll *a* were measured with a multisampling probe (RBR Ltd, Ottawa, ON, Canada) at 1, 5, 10, 15, 20, 25, 30, 40, 50, 60, 85, and 100 m depth. Samples for nitrate and total ammonium (NH_4_^+^ + NH_3_) concentrations were taken with 10-L Niskin bottles at 1, 5, 10, 15, 20, 25, 30, 40, 50, 60, 85, 110 and 130 m depth, filter-sterilized (0.20 μm, Chromafil® GF/PET-20/25, VWR, Vienna, Austria), and stored at −20°C until further analysis. Nitrate was measured by ion chromatography (S150 Chromatography System, SYKAM) using an anion exchange column at 30°C (SykroGel-AX 300 AB01 3 × 150 mm, Sykam, Germany), 4 mM Na_2_CO_3_ and 0.025 mM NaSCN as eluent, a flow rate of 1 ml min^−1^, and a conductivity detector. Total ammonium was measured fluorometrically by the ortho-phthaldialdehyde method [52]. The term total ammonium is used to refer to the sum of NH_3_ + NH_4_^+^ throughout the manuscript. DIC concentrations were analyzed after acidification [53] using cavity ring-down spectroscopy (G2201-i coupled to a Liaison A0301, Picarro Inc., Santa Clara, USA, connected to an AutoMate Prep Device, Bushnell, USA). Briefly, 1 ml subsamples of ZnCl₂-preserved lake water were injected into N₂-flushed exetainers. For samples containing ^13^C-DIC, samples were diluted with 1 ml of 2 mM NaHCO₃ because of the high ^13^C-labeling percentage (see below). Headspace overpressure was released using a needle. Subsequently, 100 μl of 20% phosphoric acid was added to convert all DIC to CO₂. Samples were incubated overnight before analysis against acidified NaHCO₃ standards.

For single cell quantification, water samples of 50 ml were fixed overnight at 4°C with paraformaldehyde (final concentration 1%, without methanol, Electron Microscopy Sciences, Hatfield, PA, USA). After fixation, cells were filtered onto 0.22 μm polycarbonate filters (GTTP, Merck Millipore, Burlington, MA, USA) and washed with 0.1 μm (Nalgene™ Rapid-Flow™, PES membrane, Thermo Fischer Scientific) filter-sterilized lake water. Filters were stored at −20°C until further analysis. AOA were quantified by catalyzed reporter deposition–fluorescence *in situ* hybridization (CARD-FISH) using the *Nitrososphaerota*-specific, horseradish peroxidase-labeled probe Thaum726 [GCTTTCATCCCTCACCGTC] and unlabeled competitors [Thaum726_compA: GCTTTCGTCCCTCACCGTC, Thaum726_compB: GCTTTCATCCCTCACTGTC]) [54, 55]. Overall picoplankton was quantified by counterstaining with 4′,6-diamidino-2-phenylindole (DAPI). Both procedures were described in detail before [26].

### Bulk and single-cell rate measurements

Potential ammonia oxidation, ammonia assimilation and dark DIC fixation were measured across seasons (Supplementary Fig. 1) using ^15^N-NH_4_^+^ and ^13^C-bicarbonate tracer incubations. Water from below the thermocline (15–30 m) and the hypolimnion (60 and 85 m) was collected with a Niskin bottle and incubated in acid-washed 4.5 l (thermocline, 85 m) or 1.8 l (60 m) glass bottles (Schott AG, Mainz, Germany), wrapped in black plastic for light protection and sealed with butyl rubber stoppers (Glasgerätebau Ochs, Bovenden-Lenglern, Germany). Rubber stoppers were cleaned beforehand overnight with 0.3 M oxalic acid and thereafter boiled for 10 min in 0.1 M sodium hydroxide. Before, in between, and after these treatments, rubber stoppers were washed and autoclaved three times in ultrapure Milli-Q® water (Sigma-Aldrich, Taufkirchen, Germany). Within 3 h after sampling, water was brought to the laboratory and amended with 5 μM ^15^NH_4_Cl and 1.3 mM NaH^13^CO_3_ (Sigma-Aldrich) to yield ^15^N and ^13^C isotopic enrichments of >90% and 35%–57%, respectively. In addition, 5 μM Na^14^NO_2_ (Sigma-Aldrich) was added to capture the ^15^N-label converted by ammonia oxidizers. Incubations were done in biological duplicates (July 2021–January 2022) or triplicates (March–September 2022). Two thermocline replicates in September 2022 failed. Incubations were done for 48 h in the dark near *in situ* temperatures: 5°C (60/85 m) and 10°C (thermocline), except for July 2021 (12°C) and January 2022 (5°C) for the latter. All samples from March 2022 were incubated at 1°C instead of anticipated 5°C (incubator error). Samples for temperature manipulation experiments were taken on the 22.02.2024 (dd.mm.yyyy) at 85 m and incubated in a temperature gradient of 5–28°C for potential ammonia oxidation rates and 5–20°C for DIC fixation and ammonia assimilation rates.

For potential ammonia oxidation rate measurements, 10 ml water were subsampled directly after tracer addition for ^15^N-ammonium labeling percentage. Added ^15^N-ammonium was measured by conversion to N_2_ using alkaline hypobromite as described previously [26, 56]. Potential ammonia oxidation rates were determined by trapping produced ^15^N-labeled nitrite in the background of added ^14^N-labeled nitrite over a period of 48 h. Here, 10 ml were subsampled with a syringe from each incubation at 4–6 time points throughout the 48 h incubations, filter-sterilized (0.20 μm, PES membrane, Sarstedt AG & Co. KG, Nümbrecht, Germany), and frozen at −20°C until further analysis. ^15^N-nitrite was converted to N_2_ using sulfamic acid [57] or to N_2_O using azide [58]. Thereafter, the isotopic composition of produced N_2_ was measured by gas chromatography isotope ratio mass spectrometry (IRMS) using an Isoprime Trace Gas for cryogenic concentration of gases coupled to a sector field IsoPrime100 with a multicollector for simultaneous detection of multiple masses (Isoprime, Manchester, UK). Potential ammonia oxidation rates were inferred from a linear regression of ^15^N-nitrite increase over time; only slopes that were significantly different from zero are reported (*P* < .05, one-tailed *t*-distribution test).

Potential dark DIC fixation and ammonia assimilation was determined in thermocline and 85 m samples obtained on 24.03.2022, 15.06.2022, and 08.09.2022 (dd.mm.yyyy) using the same incubations as for ammonia oxidation rate measurements. DIC concentrations averaged 1.6 ± 0.4 mM (range 0.7–2.2) and were measured as described above. Added ^13^C-bicarbonate used for the incubations resulted in 43.0 ± 6.0 (range 34.6–57.2) ^13^C-DIC labeling percentage. ^13^C-DIC labeling percentage was determined from unfiltered 5.9 ml subsamples taken from each incubation after addition of tracers. Samples were filled bubble-free into 5.9 ml exetainers (Labco Limited, Lampeter, UK) and preserved with saturated ZnCl_2_ solution (5 mM final concentration). After 48 h, endpoint measurements of dark ^13^C-bicarbonate and ^15^N-ammonium uptake into biomass were taken by filtering 2 to 4 l of incubated water on precombusted (450°C, 4 h) glass fiber filters (GF-75, Advantec MFS Inc., Dublin, CA, USA) using pressurized air and stainless-steel pressure tanks (AEB Kegs, Vimercate, Italy). The volume filtered depended on the biomass contained in the different water samples. After filtration, the retained biomass on GF-75 filters was rinsed with 0.1 μm (Nalgene™ Rapid-Flow™, PES membrane, Thermo Fisher Scientific, Waltham, MA, USA) filtered-sterilized lake water without isotope additions to remove unbound isotope label. GF-75 filters were then stored frozen at −20°C until further analysis. Before analysis, filters were incubated over fuming HCl to remove remaining bicarbonate and analyzed by an element analyzer (Thermo Flash EA, 1112 Series, Thermo Finnigan, Dreieich, Germany) coupled to a continuous-flow isotope ratio mass spectrometer (Delta Plus Advantage, Thermo Finnigan). To calculate bulk dark DIC and ammonium assimilation rates, obtained isotope ratios were compared to ratios obtained at T_0_ just after the addition of isotopes.

Stable isotope incorporation at the single cell level was followed by nano secondary ion mass spectrometry (nanoSIMS) [26, 59] as described in detail in Supplementary Methods. A two-sided Mann–Whitney U test (also known as the Wilcoxon rank-sum test) was used to assess differences in single-cell ^13^C- and ^15^N-enrichment between experimental groups. P-values were corrected for multiple testing using the Benjamini–Hochberg method. Statistical testing was conducted within the R core package [60].

### Metagenomics and metatranscriptomics

Metagenomic and metatranscriptomic data from Wallhausen [NCBI BioProject PRJNA691101, 26] were evaluated with a gene-centric focus on DIC fixation pathways. In brief: Nine metagenomes were obtained from 85 m depth between November 2017 and December 2018. Six metatranscriptomes were obtained from 85 m between November 2018 and November 2019. Sampling, DNA and RNA extraction, and sequencing are detailed in Klotz et al [26]. Raw Illumina reads were quality controlled and trimmed using Trimmomatic v0.38 [61] and fastx toolkit v0.0.14 (github.com/agordon/fastx_toolkit). Metagenomes were individually assembled using metaSPAdes v3.11.1 in—careful mode [62] and further processed using DRAM v1.2.3 [63] including annotation as based on KEGG [64]. DRAM was run with default parameters with contig size set to 500 nt for partial gene capture. Each *cbbL*, *aclA*, and *accB* DRAM hit was validated by BLASTp [65] against the NCBI nr (https://blast.ncbi.nlm.nih.gov/), TrEMBL and SwissProt databases [66]. Phylogenetic affiliations were resolved by aligning deduced amino acid sequences with close relatives using Mafft v7.453 [67]. Alignments were manually curated in ARB v. 6.0.3 [68] and maximum likelihood trees constructed in IQ-tree [69]. Gene coverage in metagenomes was quantified with bbmap v38.73 [70] allowing perfect and semiperfect (tolerating N in references) mappings. Transcriptional activity was inferred by mapping quality-controlled metatranscriptomic reads against target genes using bowtie2 v2.30 [71] allowing only exact matches (−-very-sensitive flag).

## Results

### Potential ammonia oxidation rates are highest below the thermocline and stable in the deep

**Figure 1:**
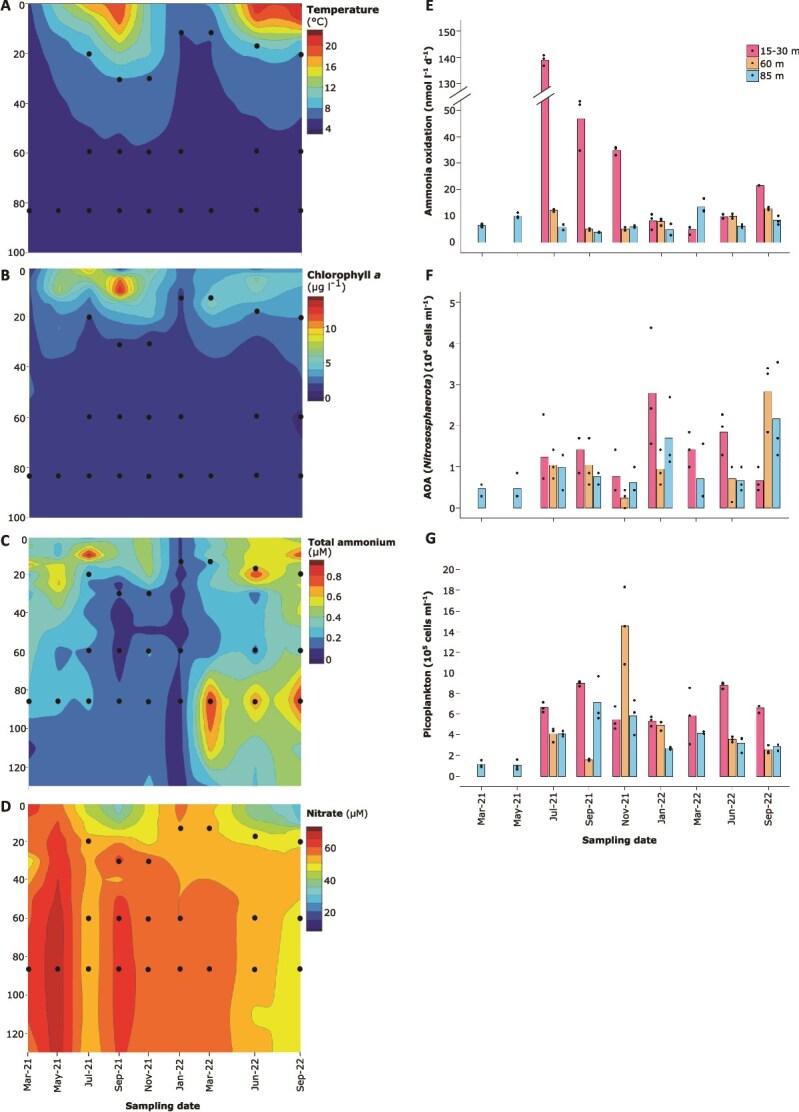
Spatiotemporal changes of environmental parameters (A–D) and corresponding dynamics of potential ammonia oxidation rates, AOA (*Nitrososphaerota*) and overall picoplankton populations (E–G) at sampling station Wallhausen in the Northwestern branch of Upper Lake Constance. Throughout the major primary productivity periods in 2021 and 2022, temperature (A) and chlorophyll *a* (B) were recorded from 1 to 100 m depth and total ammonium (NH_3_ + NH_4_^+^) (C) and nitrate (D) from 1–130 m depth. Sampling depths and dates used to determine potential ammonia oxidation rates, AOA and picoplankton populations are indicated by black dots throughout Figs 1A–D and correspond to depths below the thermocline (15–30 m) and the central hypolimnion (60 m and at 85 m). For March and May 2021, only samples from 85 m were analyzed. For March 2022, only samples below the thermocline and at 85 m were analyzed. Bars indicate the mean of individual replicates; the latter are indicated by black dotes.

Depth-resolved potential ammonia oxidation was followed at the long-term ecological research station of the University of Konstanz (station Wallhausen) throughout primary productivity periods in 2021–2022. The focus was on the hypolimnion as the major habitat of lake nitrifiers [30]. Both years showed typical spring-to-autumn thermal stratification (Fig. 1A), with phytoplankton blooms in the upper 20 m (Fig. 1B) and opposing depth gradients of total ammonium (NH_3_ + NH_4_^+^, decreasing) and nitrate (increasing) towards the hypolimnion (Fig. 1C and D ). In 2022, local deep-water ammonia maxima and a generally lower primary productivity was observed at the chosen sampling time points as compared to 2021. Winter turnover homogenized all gradients (Fig. 1).

Potential ammonia oxidation was highest below the thermocline and varied with seasons (Fig. 1E). Highest rates were observed in July 2021 (139.0 ± 2.0 nmol l^−1^ d^−1^, *n =* 3), gradually declined to 4.7 ± 1.6 nmol l^−1^ d^−1^ (*n =* 3) in March 2022 and increased again to 21.5 nmol l^−1^ d^−1^ (*n =* 1) in September 2022 (Fig. 1E). Rates at 60 and 85 m were lower and more stable, averaging 8.7 ± 3.3 nmol l^−1^ d^−1^ and 7.5 ± 3.2 nmol l^−1^ d^−1^ (*n =* 9), respectively. Parallel quantification of AOA (*Nitrososphaerota*) revealed similar abundances throughout all analyzed depths and time points averaging 1.16 ± 0.88 × 10^4^ cells ml^−1^; albeit slightly elevated population densities below the thermocline were indicated (Fig. 1F). However, AOA counts poorly correlated with potential ammonia oxidation rates (Pearson’s *r* = 0.03, *P* = .89). This was mainly due to the strong variability in potential ammonia oxidation rates below the thermocline in comparison to rather stable AOA populations (Fig. 1E and F), indicating that occasional blooms of AOB might contribute to this process at this water depth as observed by us before [30]. Overall picoplankton abundance was one order of magnitude higher than AOA and generally declined with depth (Fig. 1G).

### Potential dark dissolved inorganic carbon fixation and ammonia assimilation rates decline towards deep waters

**Figure 2:**
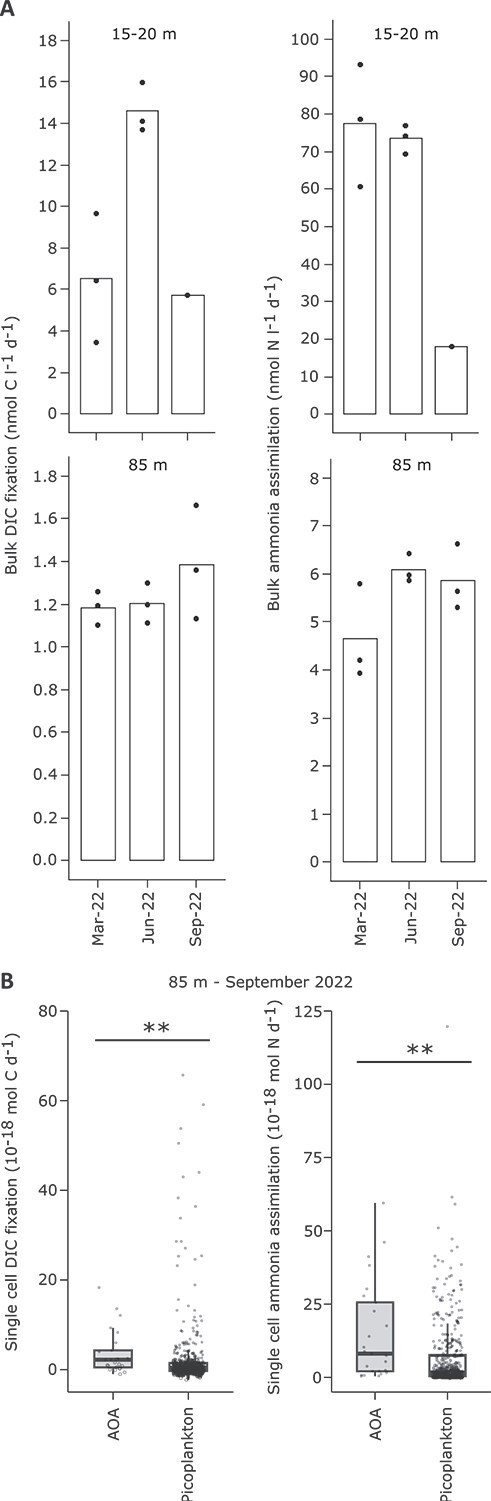
Potential dark DIC fixation and total ammonia assimilation at the bulk (A) and single cell (B) level at the sampling station Wallhausen in the Northwestern branch of Upper Lake Constance. Bulk rates were determined below the thermocline (15–20 m) and in the central hypolimnion (85 m) throughout the major primary productivity period in 2022. Bars indicate the mean of individual replicates; the latter are indicated by black dotes. Single cell rates (B) of ammonia oxidizing archaea (AOA) affiliated to the phylum *Nitrososphaerota* (*n =* 25) and the total picoplankton AOA excluding (*n =* 427) were determined for water sampled in September 2022 at 85 m depth. Box plots indicate the interquartile range (IQR) and the median. Whiskers represent a distance of 1.5 times the IQR. Circles represent measurements of individual cells. Only single cell measurements with an associated Poisson error < 5% were included. Significance values (*P*) of a pairwise Mann–Whitney U test between AOA and picoplankton cells are depicted as: * <.05; ** <.01; *** <.001.

Bulk dark DIC fixation and ammonia assimilation rates were determined below the thermocline (15–20 m) and at 85 m in 2022. Potential DIC fixation was 5–12-fold higher below the thermocline as compared to 85 m, peaking in June at 14.6 ± 1.2 nmol l^−1^ d^−1^ (*n =* 3) and dropping to 6.5 ± 1.2 nmol l^−1^ d^−1^ (*n =* 3) in March and 5.7 nmol l^−1^ d^−1^ (*n =* 1) in September (Fig. 2A). At 85 m, rates remained stable throughout all timepoints with an average of 1.3 ± 0.2 nmol l^−1^ d^−1^, *n =* 9 (Fig. 2A). Potential ammonia assimilation exceeded potential DIC fixation on average by 4.5-fold (± 0.7) at both depths (Fig. 2A). Below the thermocline, ammonia assimilation was 3–12 higher than at 85 m depth, with equally high rates in March (77.5 ± 16.3 nmol l^−1^ d^−1^, *n =* 3) and June (73.5 ± 3.9 nmol l^−1^ d^−1^, *n =* 3) and a considerable drop in September (18.0 nmol l^−1^ d^−1^, *n =* 1). At 85 m, rates were again stable, averaging 5.5 ± 0.9 nmol l^−1^ d^−1^, *n =* 9 (Fig. 2A).

Single-cell nanoSIMS analysis for a September sample at 85 m was used to validate bulk measurements. Single-cell DIC fixation among picoplankton excluding AOA (*n =* 427) showed a wide, right-skewed distribution (skewness = 5.0), with a median of 0.36 × 10^−18^ mol C cell^−1^ d^−1^ (IQR: 1.51 × 10^−18^) (Fig. 2B). In comparison, AOA cells (*n =* 25) incorporated significantly more DIC (Mann–Whitney U test, *P* = .002). In particular, AOA showed less skew (1.7) and a higher median of 2.17 × 10^−18^ mol C cell^−1^ d^−1^ (IQR: 3.8 × 10^−18^) (Fig. 2B). Extrapolating combined picoplankton and AOA single-cell rates to total DAPI counts yielded 0.78 nmol C l^−1^ d^−1^, which was slightly lower but in the same range as bulk DIC fixation rates (Fig. 2B). This is likely due to isotope dilution by the staining procedure preceding nanoSIMS analysis, which would affect CARD-FISH-stained AOA stronger than the DAPI-stained remaining picoplankton [72–74]. Extrapolation of AOA single-cell rates to the overall AOA population yielded 0.08 nmol C l^−1^ d^−1^, corresponding to 11% of total single-cell-based picoplankton DIC fixation.

Ammonia assimilation of picoplankton excluding AOA showed again a wide, right-skewed distribution (skewness = 3.6) with a median of 1.67 × 10^−18^ mol N cell^−1^ d^−1^ (IQR: 7.19 × 10^−18^) (Fig. 2B). Also here, AOA cells incorporated significantly more ammonia than the remaining picoplankton (Mann–Whitney U test, *P* < .001) with a median of 8.14 × 10^−18^ mol N cell^−1^ d^−1^ (IQR: 23.4 × 10^−18^, skewness 1.2) (Fig. 2B). Extrapolation of picoplankton and AOA single-cell rates to total DAPI counts yielded a combined ammonia assimilation rate of 1.93 nmol N l^−1^ d^−1^, which was again lower but in the same order of magnitude as compared to bulk ammonia assimilation rates (Fig. 2A). Extrapolation of AOA single-cell rates alone to the overall AOA population yielded 0.39 nmol N l^−1^ d^−1^, corresponding to 17% of total single-cell-based picoplankton ammonia assimilation. Since isotope dilution factors caused by CARD-FISH and DAPI staining are different for ^13^C- and ^15^N-enrichment, vary across taxa [72–74], and are hard to assess in environmental settings, this data was not evaluated in detail in respect to N:C fixation ratios. For AOA, a brief discussion is provided in Supplementary Material.

### Potential ammonia oxidation, dark dissolved inorganic carbon fixation, and ammonia assimilation differ in temperature dependency

**Figure 3:**
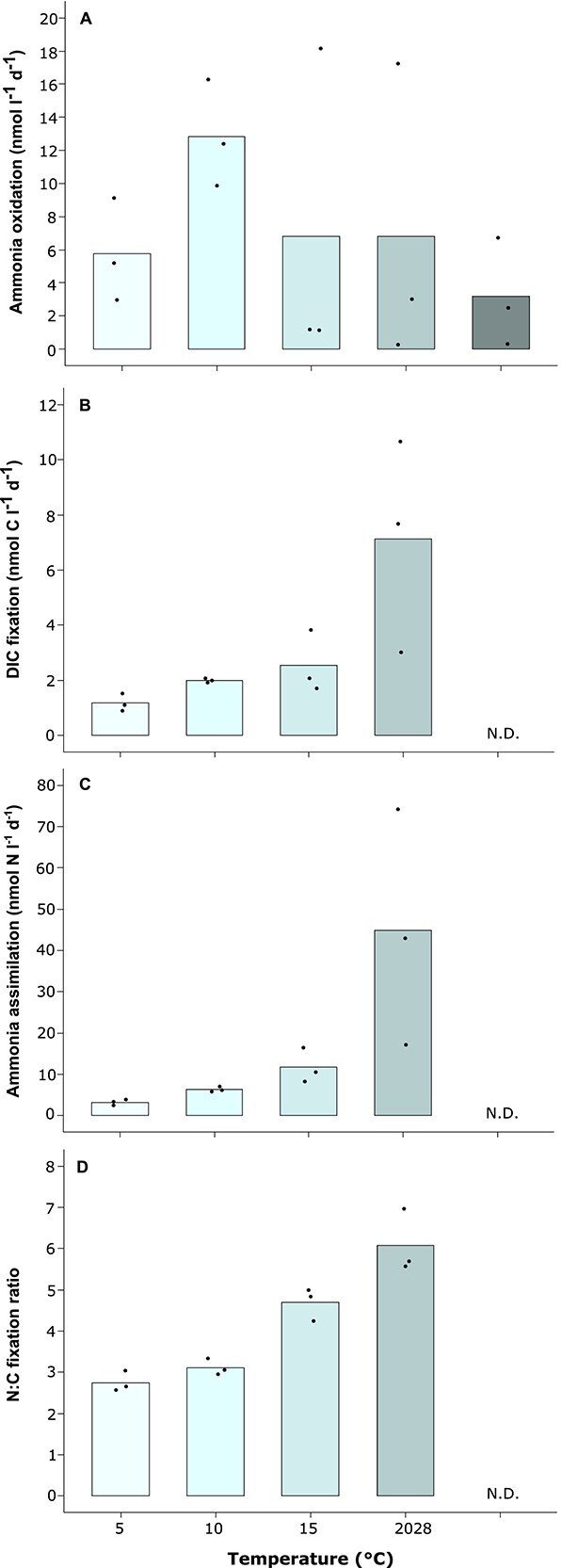
Temperature dependency of potential ammonia oxidation (A), DIC fixation (B), total ammonia assimilation (C), and the ratio of ammonia assimilation to DIC fixation (D) at the bulk level. Water sampled at 85 m depth in February 2024 was incubated in biological triplicates along a temperature gradient of 5 to 28°C (ammonia oxidation) or 5 to 20°C (DIC fixation and total ammonia assimilation). Bars indicate the mean and dots individual biological replicates. N. D.—not determined.

To assess the temperature response of potential ammonia oxidation, DIC fixation, and ammonia assimilation, water from 85 m depth was incubated across 5–20°C. For ammonia oxidation, 28°C was tested in addition but had to be left out for experimental capacity reasons for DIC fixation and ammonia assimilation. The sampled water contained 2.96 ± 0.38 × 10^5^ picoplankton cells ml^−1^, including 0.24 ± 0.17 × 10^5^ AOA cells ml^−1^ (*n =* 5). Potential ammonia oxidation rates doubled from 5 to 10°C but declined again at higher temperatures back to levels observed at 5°C, albeit with high variability at 15 and 20°C (Fig. 3A). Nevertheless, also at 15 and 20°C two out of three replicates showed a clear decline in rates (Fig. 3A). In contrast, potential DIC fixation and ammonia assimilation increased exponentially with higher temperature (Fig. 3B and C). Interestingly, the ratio of ammonia to DIC incorporated at the bulk level into biomass increased exponentially as well ranging from 2.9 ± 0.2 at 5°C to 6.5 ± 0.7 at 20°C (*n =* 3 each; Fig. 3D). However, both processes are not necessarily strictly connected since heterotrophic picoplankton not involved in DIC fixation could have used part of the ammonia as N source for biomass production.

### Dissolved inorganic carbon fixation pathways are actively expressed by a wide range of microorganisms

Nine metagenomes from 85 m depth (spanning one year) were screened for *accB*, *aclA*, and *cbbL* as marker genes for the 3HP/4HB, rTCA, and CBB cycles, respectively. Each metagenome contained a single *accB* variant, which was 100% identical to *accB* of *Cand.* N. limneticus [26] and clustered with AccB of other *Nitrosopumilus* species (Supplementary Fig. 2). Retrieved *aclA* formed two distinct *Nitrospira* clusters, with one hit per metagenome each. Within-cluster amino acid sequences were identical and differed by 0.9% between clusters. Both AclA types were most closely related to AclA of *Nitrospira lenta* BS10 (94.7 and 95.6% identity; Supplementary Fig. 3). Retrieved *cbbL* represented 45 variants (Supplementary Fig. 4), which all had >80% deduced amino acid sequence identity to the large subunit of validated RubisCO form IA or IC (SwissProt). Form IA CbbL (14 hits) were affiliated with *Nitrosospira* AOB and *Comamonadaceae* (*Betaproteobacteria*) as well as *Synechococcus* and *Cyanobium* (*Cyanobacteriota*). Form IC CbbL (31 hits) were affiliated with *Nitrotoga* NOB, *Comamonadaceae,* and *Methylophilaceae* (*Betaproteobacteria*), *Mycobacteriaceae* (*Actinomycetota*), uncultured *Alphaproteobacteria*, and cryptophytes (protists) related to *Teleaulax* (Supplementary Fig. 4). Details are given in Supplementary Results.

**Figure 4:**
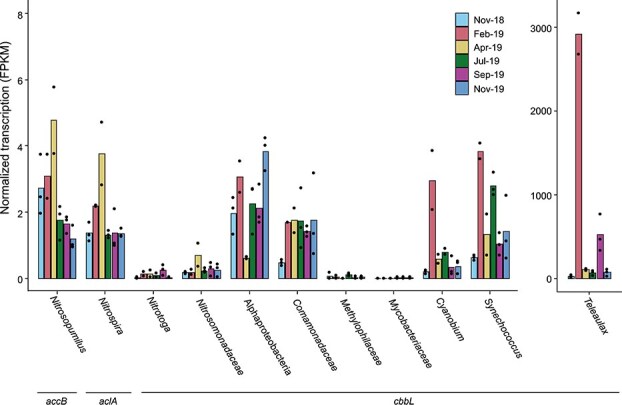
Seasonal dynamics in transcription of hallmark genes of DIC fixation pathways at 85 m depth in Lake Constance, sampling station Wallhausen. Transcriptional activity of 3HP/4HB-encoding microorganisms is represented by *accB* transcripts, of rTCA-encoding microorganisms by *aclA* transcripts, and of CBB cycle-encoding microorganisms by *cbbL* transcripts, respectively. Transcription is displayed as FPKM (normalized transcription in fragments per kilobase million). The bars represent the mean of three replicates (except for February and April 2019 with two replicates). Dots display individual replicates. Please note the two different scales of the y-axes.

Across metagenomes, *Nitrosopumilus accB* had the highest relative abundance (16.9 ± 13.2 reads per kilobase of gene per gigabase of metagenome, RPKG), followed by *Nitrospira aclA* (4.3 ± 1.2 RPKG) and all *cbbL* combined (4.3 ± 1.5 RPKG), with *Comamonadaceae* as the dominant *cbbL*-encoding group (Supplementary Fig. 5). Since the hypolimnion is a very stable environment with little variation in picoplankton community structure [30] and DIC fixation activity (see above), transcriptional activity was followed by metatranscriptomics in the follow-up year (Fig. 4). At 85 m, *Teleaulax cbbL* transcripts dominated with a median of 92 fragments per kilobase of transcripts per million mapped reads (FPKM), ranging from 29 to 2917 FPKM (skewness 2.3). All other DIC fixation marker genes showed at least 1–2 orders of magnitude lower transcription. Transcription of *Nitrosopumilus accB*, *Nitrospira aclA*, and *cbbL* of *Alphaproteobacteria*, *Comamonadaceae*, *Synechococcus*, and occasionally *Cyanobium* ranged from 0.4 to 5.8 FPKM (Fig. 4). *Nitrosomonadaceae* and *Nitrotoga cbbL* had even lower transcriptional activity (median of 0.2 and 0.1 FPKM, respectively), while *Methylophilaceae* and *Mycobacteriaceae* showed negligible expression (0.00–0.11 and 0.00–0.01 FPKM, respectively). Overall, transcriptional activity patterns were stable across the year, with no consistent seasonal trends, apart from exceptionally high *Teleaulax cbbL* transcriptional activity in February (2917 FPKM, *n =* 2) and September 2019 (526 ± 224 FPKM, *n =* 3). Interestingly, *Nitrosopumilus accB* and *Nitrospira aclA* transcript levels were strongly correlated (Pearson’s *r* = 0.92, *P* = .01), as were *Nitrosomonadaceae cbbL* and *Nitrospira aclA* (Pearson’s *r* = 0.88, *P* = .02).

## Discussion

### Decoupled dynamics of potential ammonia oxidation and dark dissolved inorganic carbon fixation

We assessed spatiotemporal dynamics of potential ammonia oxidation and dark DIC fixation in the hypolimnion of deep oligotrophic Lake Constance and there *in situ* temperature dependencies. During thermal stratification, both processes varied across seasons below the thermocline but remained stable, albeit at considerably lower rates, at the center of the hypolimnion (Fig. 1E and 2A). The same was true for potential ammonia assimilation as a more general activity marker for growth [75, 76] (Fig. 2A). At the hypolimnion center of Lake Constance, potential dark DIC fixation was ~10 times lower than in deep, aphotic waters of oligo-mesotrophic Lake Maggiore [10] and ultraoligotrophic Lake Superior [29], but comparable to oligotrophic Lake Taupo [77]. In contrast, potential ammonia assimilation was well comparable to rates in deep aphotic waters of Lake Superior [76]. Ammonia oxidation has been rarely determined in deep oligotrophic lakes [78], with measurements via ^15^N-labeling being even more limited [26, 29, 79]. Apart from Lake Constance, only Lake Superior has been thoroughly studied, showing slightly higher but comparable ammonia oxidation rates [29, 79]. Although urea and cyanate could not be tested as alternative substrates for ammonia oxidizers, marine studies in coastal and shelf regions showed that ammonia-driven oxidation rates are typically one order of magnitude higher than those driven by urea or cyanate [59, 80, 81], with higher contributions of urea reported rather for polar regions and the open (oligotrophic) ocean [80–84].

During thermal stratification, the lower thermocline of Lake Constance marked the intersection of total ammonium (NH₃ + NH₄^+^) and nitrate counter-gradients (Fig. 1). Higher ammonia availability and reduced competition for ammonia, due to declining populations of photoautotrophs (Fig. 1B) and heterotrophic bacterioplankton (Fig. 1G), likely contributed to elevated ammonia oxidation rates below the thermocline relative to the center of the hypolimnion. In addition, two-fold higher temperatures (Fig. 1A) likely further enhanced activity of both, potential ammonia oxidation and dark DIC fixation [85, 86]. Interestingly, spatiotemporal dynamics of potential ammonia oxidation and dark DIC fixation were not correlated (Pearson’s *r* = 0.11, *P* = 0.69), as were potential ammonia oxidation and assimilation (Pearson’s *r* = −0.25, *P* = 0.35). In 2022, potential dark DIC fixation was 5–12-fold higher below the thermocline compared to the hypolimnion center (85 m), peaking in June (Fig. 2A), whereas potential ammonia oxidation was even lower (March) or only 1.6–2.6-fold higher below the thermocline, with the maximum in September (Fig. 1E).

To isolate temperature effects, we incubated water from the hypolimnion center across 5–20/28°C (Fig. 3). Potential ammonia oxidation rates peaked near 10°C, matching temperature optima observed for AOA-dominated ammonia oxidation in the ocean [85]. In the central hypolimnion of Lake Constance, the AOA *Cand.* N. limneticus has been shown to dominate ammonia oxidation by far [26, 30]. Combined with our data, this indicates that this widespread and extremely slowly evolving AOA [21] is cold-adapted. In contrast, potential dark DIC fixation and ammonia assimilation increased exponentially across the tested temperature range. In coastal systems, similar increases have been documented with DIC fixation temperature optima ranging from 22–32°C [86]. We did not measure additional environmental parameters that could be related to measured activities. However, standard monitoring at the center of Lake Constance conducted by the Institute for Lake Research (LUBW State Institute for Environment Baden-Württemberg), showed negligible variation for O_2_ (10.2 ± 1.0 mg l^−1^) and chloride (7.3 ± 0.3 mg l^−1^) in the water body spanning the water column below the thermocline to the central hypolimnion throughout the study period [51]. Only orthophosphate varied across seasons peaking in January 2022 (2.93 ± 0.38 μg l^−1^), steadily declining until October 2022 (0.13 ± 0.05 μg l^−1^), and increasing again until December 2022 (1.48 ± 0.42 μg l^−1^) [51]. However, there was little spatial variation in orthophosphate along the water column below the thermocline to the central hypolimnion per sampling time point [51] that could potentially explain observed differences in potential ammonia oxidation and dark DIC fixation along this spatial gradient (Fig. 1E, Fig. 2A). In summary, the observed spatiotemporal mismatch in measured ammonia oxidation (Fig. 1E) and dark DIC fixation activities (Fig. 2A) combined with the results from our temperature manipulation experiment (Fig. 3) indicate that decoupled activity changes of both processes are driven by temperature differences.

### AOA contribute 11% to dark DIC fixation in Lake Constance

Nitrifer contributions to dark DIC fixation can be estimated based on the amount of nitrogen (NH_3_ or NO_2_^−^) oxidized per mol of carbon fixed into biomass. In Lake Constance, AOA dominate the nitrifying community in the hypolimnion [26, 30], consistent with findings in other deep oligotrophic lakes [13, 21–25, 27]. In culture, AOA oxidize ~10–11 N per mol C fixed [87, 88]. Applying this ratio to our measured potential ammonia oxidation rates at the center of the hypolimnion (Fig. 1E) would suggest that AOA-driven dark DIC fixation accounts for ~50% of total potential dark DIC fixation. However, ^13^C-DIC incorporation into single AOA cells (Fig. 2B) indicated a much lower contribution to dark DIC fixation of ca. 11% when extrapolated to total AOA abundance. This represents a conservative estimate, as CARD-FISH prior to nanoSIMS can dilute isotopic signals by 16%–77% [72–74]. Even if considering the highest isotopic dilution reported, AOA would not exceed 19.5% of dark DIC fixation, supporting the lower estimate. This aligns well to the share of AOA-driven dark DIC fixation reported for Lake Maggiore [10]. Also in the Atlantic, AOA where shown to incorporate 10 times less ^14^C-DIC than bacterial picoplankton [89] and in the eastern tropical Pacific AOA fixed only half as much C into biomass per NH₃ oxidized as compared to pure cultures [90], indicative of a lower metabolic efficiency under environmental settings. In the hypolimnion of Lake Constance, this could be caused by the low temperature (5°C) that substantially deviates from the determined ammonia oxidation optimum at 10°C (Fig. 3), possibly necessitating a higher energy demand for maintenance as compared to growth. Additional numerically relevant nitrifiers involved in dark DIC fixation would be mainly represented by *Nitrospira* NOB in Lake Constance [26, 30]. Since their DIC fixation yield in pure culture is considerably lower (ca. 28 NO_2_^−^ oxidized per 1 mol C fixed) [88] and they are one order of magnitude less abundant than AOA [26, 30], their contribution to overall DIC fixation in the hypolimnion of Lake Constance can be regarded to be well below 10%.

Our meta-omics data corroborated that, beyond the dominant AOA populations at depth (Fig. 1F), a broader range of microorganisms must have substantially contributed to dark DIC fixation in the cold hypolimnion (Fig. 2B). To resolve their identity, we integrated metagenomic and metatranscriptomic approaches. Among DIC-fixation pathway encoding microorganisms, chemolithoautotrophic nitrifiers made up the largest share at the center of the hypolimnion, followed by CBB-encoding *Alphaproteobacteria*, *Betaproteobacteria*, actinomycetes, cyanobacteria, and cryptophytes related to *Teleaulax* (Supplementary Fig. 5). Transcriptional activity (measured the following year) differed substantially from relative abundance patterns. *Teleaulax* transcripts clearly dominated (Fig. 4), although longer mRNA half-lives in protists (>30 min to 144 h) [91, 92] as compared to pelagic prokaryotes (9 min to >6 h) [93, 94] may skew this comparison. Transcriptional activities of the AOA *Cand*. N. limneticus, putative NOB related to two *Nitrospira* spp. and of unclassified *Alphaproteobacteria*, *Comamonadaceae* (*Betaproteobacteria*), and the cyanobacterial *Cyanobium* and *Synechococcus* spp. were comparable, constituting jointly the second most transcriptionally active group.

While transcriptional activity does not equal metabolic rates, it allows to identify potentially relevant microorganisms. Our metatranscriptomic data corroborated that AOA and other nitrifiers contributed only in parts to dark DIC fixation, besides dominating numerically among DIC-fixation pathway encoding microorganism in Lake Constance (Supplementary Fig. 5) and other deep oligotrophic lakes [12, 13, 21, 23, 27–29]. Cryptophytes and cyanobacteria (*Cyanobium* and *Synechococcus*) are typically active in the epilimnion but showed residual transcriptional activity in the hypolimnion that exceeded that of all nitrifiers (Fig. 4). In February, water column mixing under isothermal conditions [26] may have transported photosynthetically active microorganisms downward, explaining peak transcript levels in the hypolimnion (Fig. 4). However, even during thermal stratification, cryptophyte and cyanobacteria transcriptional activities remained higher or comparable to those of nitrifiers at the center of the hypolimnion (Fig. 4).

Bacterivorous, aplastidic cryptophytes constitute about two thirds of heterotrophic nanoflagellates (HNF) across diverse freshwater lakes that broadly differ in trophic state [95]. Their small size relative to chloroplast-bearing counterparts [95] explains their detection among picoplankton in our study (0.2–5.0 μm). In Lake Constance, they account for ca. 60 and 80% of HNF in the hypolimnion and epilimnion, respectively, with densities of several hundred cells ml^−1^ [95]. Our data indicates that they still encode RubisCO, as known from other non-photosyntethic cryptophytes [96], and apparently activate their CBB cycle in the dark (Fig. 4). Similarly, picocyanobacteria can grow mixotrophically on diverse organic compounds [97] or as facultative heterotrophs [98]. Indeed, stable *Synechococcus* populations of 10^3^ cells ml^−1^ were detected down to 1000 m in the Black Sea, with viable representatives at 750 m that encode genomic traits for heterotrophy [99, 100]. Since the CBB cycle can support intracellular redox balance and occurs in heterotrophic microorganisms [32, 46], its transcriptional activation by cryptophytes and picocyanobacteria (Fig. 4) likely contributed to dark DIC fixation in Lake Constance. The same applies to *Comamonadaceae* (*Betaproteobacteria*) and unclassified *Alphaproteobacteria* that transcriptionally activated their CBB cycle as well, though their energy metabolism remains speculative at present (see Supplementary Discussion). Finally, carboxylating reactions in the metabolism of heterotrophic bacteria [47] may have contributed considerably to dark DIC fixation, as shown for marine systems [101, 102].

## Conclusion

We assessed the current status of two biogeochemical processes involved in C and N cycling, dark DIC fixation and ammonia oxidation, in Lake Constance as an important model lake for deep oligotrophic freshwaters worldwide and analyzed how both are interconnected. Our data shows that both biogeochemical processes get decoupled with increasing temperatures despite the general assumption that they are strongly linked by the activity of large standing populations of chemolithoautrophic *Nitrososphaerota* AOA in the hypolimnion. Similar to findings in the deep aphotic part of the ocean [90, 102, 103], we show that this disconnect is due to AOA driving only a minor part of dark DIC fixation, with the majority being attributed to metabolically and taxonomically diverse picoplankton. Given that freshwater lakes are increasingly affected by global warming [1–3] including Lake Constance (Supplementary Fig. 6), and deep oligotrophic lakes store the majority of Earth’s lake water, our results provide important parameters to integrate these microbial temperature responses into models of lake biogeochemistry including climate change scenarios.

## Supplementary Material

Bosviel_et_al_2025-10-02-supplements_ycaf201

## Data Availability

The metagenomics and metatranscriptomics datasets analyzed in the current study are available in the National Center for Biotechnology Information (NCBI) under BioProject number PRJNA691101 (https://www.ncbi.nlm.nih.gov/bioproject/PRJNA691101/).
