# Supplementary material for: Spatiotemporal and temperature-dependent disconnect between ammonia oxidation and dark DIC fixation in deep oligotrophic Lake Constance
Source: ISME Commun. 2025 Nov 10;5(1):ycaf201. doi: 10.1093/ismeco/ycaf201 (PMC12642877; doi:10.1093/ismeco/ycaf201)
Supplement: Bosviel_et_al_2025-10-02-supplements_ycaf201 [file bosviel_et_al_2025-10-02-supplements_ycaf201.pdf]

Supplemental Material for

Spatiotemporal and temperature-dependent disconnect between ammonia oxidation and dark DIC fixation in deep oligotrophic Lake Constance

Jade Bosviel[1], Katharina Kitzinger[2,3], Francesca Vulcano[1], Franziska Klotz[4], Anton Legin[3], Petra Büsing[1], Thorsten Rennebarth[5], Joerdis Stuehrenberg[2], Hannah Marchant[2,6], Michael Wagner[3,7], Martin Wessels[5], David Schleheck[4], Marcel M. M. Kuypers[2] and Michael Pester[1,8*]

1. *Leibniz Institute DSMZ – German Collection of Microorganisms and Cell Cultures, Inhoffenstr. 7B, D-38124 Braunschweig, Germany*
2. *Max Planck Institute for Marine Microbiology, Celsiusstrasse 1, D-28359 Bremen, Germany*
3. *Centre for Microbiology and Environmental Systems Science, University of Vienna. Djerassiplatz 1, 1030 Vienna, Austria*
4. *Limnological Institute, Department of Biology, University of Konstanz, Universitätsstrasse 10, D-78457 Konstanz, Germany*
5. *Institute for Lake Research, LUBW State Institute for Environment Baden-Württemberg, Argenweg 50/1, 88085 Langenargen, Germany*
6. *MARUM – Centre for Marine Environmental Sciences, University of Bremen, Leobener Str. 8, Bremen, D-28359, Germany*
7. *Department of Chemistry and Bioscience, Aalborg University, Fredrik Bajers Vej 7H, 9220 Aalborg, Denmark*
8. *Technical University of Munich, School of Life Sciences, Emil-Ramann-Strasse 4, D-85354 Freising, Germany*

*Corresponding author: Michael Pester, Mail: Michael.Pester@tum.de

Supplementary materials and methods

nanoSIMS analysis

Prior to CARD-FISH targeting AOA for nanoSIMS, 5 mm diameter pieces were cut out from GTTP filters containing fixed and incubated bulk microbial communities. For determination of natural abundances of ^{13}C and ^{15}N, non-incubated bulk microbial communities from a 20-m sample from September 2022 were used. Laser markings for orientation were made using a laser microdissection microscope (Leica LMD 7000, Leica, Wetzlar, Germany). CARD-FISH was performed as described above, with the exception that filters were not embedded in agarose and OregonGreen488 tyramides were used. Visualization of AOA (targeted by probe Thaum726) and all other microorganisms (stained by DAPI)

was done on an inverted epifluorescence microscope (Thunder Imager DMi8, Leica). Prior to nanoSIMS measurements, all filters were coated with a 25 nm gold layer (K550X Emitech, Quorum Technologies Ltd., Ashford, UK) to ensure sufficient conductivity.

nanoSIMS measurements were carried out on a NanoSIMS 50L (Cameca, Gennevilliers, France) at the Large-Instrument Facility for Environmental and Isotope Mass Spectrometry at the University of Vienna. Prior to data acquisition, analysis areas were pre-conditioned *in situ* by rastering of a high intensity, defocused Cs^+ ion beam in the following sequence of high and extreme low ion impact energies (HE/16 keV and EXLIE/50 eV, respectively): HE at 100 pA beam current to a fluence of 2.5 × 10^{15} ions/cm2; EXLIE at 400 pA beam current to a fluence of 5.0E16 ions/cm2; HE to a fluence of 2.5 × 10^{14} ions/cm2. The detectors of the multicollection assembly were positioned to enable parallel detection of $^{12}C_2^-$, $^{12}C^{13}C^-$, $^{12}C^{14}N^-$, $^{12}C^{15}N^-$, $^{31}P^-$ and $^{32}S^-$ secondary ions and the mass spectrometer was tuned to achieve a mass resolving power (MRP) of >9,000 (according to Cameca's definition) for detection of C_2^- and CN^- secondary ions. All data were acquired as multilayer image stacks obtained by sequential scanning of a finely focused Cs+ primary ion beam (~1 pA allowing for physical resolution of approximately 80 nm) over areas between 25 × 25 μm^2 and 50 × 50 μm^2 with 512 × 512 pixel image resolution. The per-pixel dwell time of the primary ion beam was 1.5 ms/(pixel∗cycle).

nanoSIMS images were processed using the software Look@NanoSIMS (vs. 24-12-20 on Matlab vs. R2024b academic license). The individual images were aligned to compensate for positional variations arising from room temperature, primary ion beam and/or sample stage drift. Secondary ion signal intensities were corrected for detector dead time and quasi-simultaneous arrival effect on a per-pixel basis applying sensitivity factors of 1.06, 1.05 and 1.1 for C_2^-, CN^- and other ions, respectively. Regions of interest (ROIs), referring to individual cells, were manually defined based on the nitrogen-, phosphorus- and sulfur- secondary ion signal intensity distribution maps in correlation to the respective FISH images. Carbon isotope composition images displaying the $^{13}C/(^{12}C+^{13}C)$ isotope fraction, designated as ^{13}C-atom%, were inferred from the C_2^- secondary ion signal intensity distribution images via per-pixel calculation of $^{13}C^{12}C^-/(2*^{12}C_2^-+^{13}C^{12}C^-)*100$ intensity ratios. Accordingly, nitrogen isotope composition images displaying the $^{15}N/(^{14}N+^{15}N)$ isotope fraction (^{15}N-atom%) were calculated from $^{12}C^{15}N^-/(^{12}C^{14}N^-+^{12}C^{15}N^-)*100$ intensity ratios.

CARD-FISH has been shown to dilute the isotopic composition of target and non-target cells depending on the used CARD-FISH protocol, the cells' growth stages and types [1-3]. For our experiments, we did not account for isotopic dilution of AOA and other microorganisms, as this dilution effect cannot be well constrained for environmental samples. Thus, we potentially underestimate the ^{13}C and ^{15}N-enrichment of our single cell data and the presented estimates are conservative. Isotopic dilution by

CARD-FISH affects ^{13}C- enrichment stronger than ^{15}N-enrichment [1-3], which is why single-cell data was not evaluated for N:C fixation ratios.

Supplementary results

RubisCO form IA CbbL retrieved by metagenomics

Cyanobacterial CbbL form IA sequences clustered into three distinct groups, which were affiliated to *Synechococcus* spp. and *Cyanobium* spp. (Supplementary Fig. 4). The first cluster consisted of four identical sequences and their closest relative (100% deduced amino acid identity) was *Synechococcus lacustris* Tous, isolated from an oligotrophic water reservoir [4]. Sequences forming a second cluster were 98.6–99.6% similar to each other and were closely related to *Synechococcus* sp. MAG Baikal-G1 from Lake Baikal (98.6–99.2%) [4, 5]. The two *Synechococcus* clusters were 97.3–97.6% similar to each other on the amino acid sequence level. A third cyanobacterial cluster covered four identical CbbL sequences, which were identical to cultured *Cyanobium* sp. Tous-2 from an oligotrophic water reservoir and MAG Baikal-G2 from Lake Baikal [4, 5] (Supplementary Fig. 4).

The three betaproteobacterial sequences of CbbL form IA clustered together with sequences affiliated with the families *Comamonadaceae* and *Nitrosomonadaceae* (Supplementary Fig. 4). CbbL_95090 was identical to the RubisCO-CbbL of the AOB *Nitrosospira* sp. ON33M_5M_Binsanity_121, a MAG obtained from Lake Ontario, USA [6]. CbbL_334569 was closely related to CbbL of the AOB MAG *Nitrosospira* sp. MC4_S2_ CONCOCT_122 (99.6%) from Lake Erie, USA [6]. CbbL_334569 and 95090 were only distantly related to each other (90.4%). CbbL_45609 is identical to CbbL of a freshwater Betaproteobacterium WB5_2A_165 MAG from Chattahoochee River, USA [7] and 96.5% similar to CbbL of *Hydrogenophaga* sp. MAG CROMO_BS_ 123_1 [8] and 96.2% similar to CbbL of cultured *Acidovorax facilis* LMG 2193 (Supplementary Fig. 4). All accession numbers of listed sequences are given in Supplementary Fig. 4.

RubisCO form IC CbbL retrieved by metagenomics

RubisCO form IC CbbL affiliated with the family *Comamonadaceae* (*Betaproteobacteria*) fell into two distinct clusters (Supplementary Fig. 4). In the first cluster, the sequences CbbL_12776, CbbL_19297, and CbbL_12734 were 100% identical to each other and shared 98.3% similarity to CbbL_18463. In addition, the sequences CbbL_228851, CbbL_222920, and CbbL_230487 were part of this cluster and shared 95.2–97% similarity to the first four CbbL sequences (Supplementary Fig. 4). All seven sequences were distantly related to Cbbl of *Hydrogenophaga* sp. MAG_35 (94.2–96.6%) retrieved from a ground-water aquifer [9] and their next cultured relative *Hydrogenophaga* sp. RAC07 (93.5–95.9%) isolated from a microalga [10]. The second *Comamonadaceae* cluster of form IC CbbL consisted

of two distinct sequence types (Supplementary Fig. 4). CbbL_174948 closest relative was *Limnohabitans* MAG Go_Leine_bin_80 (92%) retrieved from wastewater [11]. The second group consisted of four nearly identical (99.7–100%) CbbL sequences closely related to a Betaproteobbacterium MAG LLD_101 (99.8%) retrieved from the Chattahoochee River, USA [7] and *Rhodoferax* sp. MAG Baikal-deep-G92 (99.6%) from the hypolimnion of Lake Baikal [5]. Three additional CbbL form IC sequences affiliated to Betaproteobacteria were detected. CbbL_17819 was closely related to a freshwater *Nitrotoga* MAG MC9_S7_M_CONCOCT_170 (98.3%) from Lake Huron, USA [6]. The remaining two CbbL clustered with members of the *Methylophilaceae.* CbbL_225974 was distantly related to *Novimethylophilus kurashikiensis* La2-4 (93.0%) and *Methylobacillus* sp. MM3 (92.9%) and CbbL_93102 was distantly related to CbbL of the freshwater MAG *Methylotenera* sp. RIFCSPLOWO2_02_FULL_45_14 (89.0%) [12] (Supplementary Fig. 4).

From nearly every metagenome a contig was recovered with a CbbL sequence clustering together with two alphaproteobacterial MAGs from deep layers of Lake Baikal (Baikal-deep-G50) [5, 13] and Lake Tanganyika (K_Offshore_80m_m2_162) [14] with similarities of 95.1% and 91.8%, respectively (Supplementary Fig. 4). The CbbL sequences themselves were identical to each other, except for partial CbbL_27956 (99.7%). No closely related cultured relative (including those with *Candidatus* status) was found with Blastp with similarities above 90%. Six identical CbbL sequences formed a cluster affiliated with two actinobacterial MAGs, which were *Mycolicibacterium* sp. SupBloom_Metag_022 from a freshwater cyanobacterial bloom in oligotrophic Lake Superior (99.4%) [15] and freshwater-originated Mycobacterium sp. UBA8140 (99.0%) [16]. In addition, a eukaryotic CbbL was detected with the photosynthetic cryptophyte *Teleaulax amphioxeia* HACCP-CR01 (99.2%) as the next cultured relative (Supplementary Fig. 4). Strong overestimation of *Teleaulax*-related protists due to polyploidy is not indicated as *Teleaulax amphioxeia* was shown to undergo dimorphism between an haploid and diploid genome depending on environmental conditions [17]. All accession numbers of listed sequences are given in Supplementary Fig. 4.

Supplementary discussion

Potential metabolism of *cbbL*-encoding microorganism

Members of the *Comamonadaceae* (Betaproteobacteria) and unclassified Alphaproteobacteria were additional groups that transcriptionally activated their CBB cycle at transcript levels well comparable to nitrifying AOA and NOB (Fig. 4). Among the *Comamonadaceae*, actively transcribed *cbbL* could be identified that were related to (putatively) facultative chemolitoautrophs of the genera *Hydrogenophaga*, *Rhodoferax,* and *Limnohabitans* with the highest share of transcripts attributed to *Hydrogenophaga*-related *cbbL* (data not shown). *Hydrogenophaga* spp. can either use DIC or organic

carbon as carbon source while gaining energy from H_2 or thiosulfate oxidation [18, 19]. Some *Rhodoferax* spp., such as *Rhodoferax antarcticus*, can switch between a photoautrophic/photoheterotrophic lifestyle under anoxic, light-exposed conditions and a heterotrophic metabolism under oxic, dark conditions, while encoding the CBB cycle and being cold adapted [20]. Furthermore, related *cbbL* detected in our study had a high sequence identity to *cbbL* of *Rhodoferax* MAG Baikal-deep-G92 (Suppl. Results), which was assembled from the hypolimnion of Lake Baikal and encoded thiosulfate oxidation pathway genes as well [5], making a chemolithoautotrophic lifestyle feasible. *Limnohabitans* spp. are typical heterotrophic freshwater bacteria [21-23]. However, genomic evidence indicated that members of this genus encode RubisCO and might be capable of a photoheterotrophic [24, 25] or chemolithoautotrophic metabolism relying on the oxidation of ammonia or reduced sulfur compounds [5, 24]. Little is known about *cbbL* -encoding *Comamonadaceae* and unclassified *Alphaproteobacteria* other than that they have been detected in the hypolimnion of other oligotrophic lakes as well [5].

Single cell N:C incorporation into AOA

Our single cell data pointed to a N:C fixation ratio in AOA cells that deviates considerably from the Redfield ratio, indicating a higher N than C incorporation on a 48-h timescale (Fig. 2b). We can only speculate about the reasons. It is known that isotope dilution factors by the CARD-FISH procedure can affect ^{13}C-enrichment stronger than ^{15}N-enrichment [1-3], albeit to a smaller extent than we observed. In addition, the negatively charged S-layer of AOA strongly binds and concentrates positively charged ammonium at the cell surface [26]. However, we consider it highly unlikely that ammonium bound solely by ionic interactions at the cell surface is not removed by the many washing steps of the CARD-FISH procedure. Interestingly, genes encoding ammonia transporters are among the highest transcribed by Cand. *N. limneticus in situ* year-round [27]. This may point to a high ammonium uptake rate and the potential incorporation of ammonia into (unknown) N-storage compounds, which could explain the high measured N-assimilation. In addition, the discrepancy between DIC and ammonia assimilation could indicate that Cand. *N. limneticus* can grow mixotrophically, utilizing organic carbon in addition to DIC for biomass growth. Alpha-keto acids have initially been proposed to support mixotrophic growth of AOA [28] but this hypothesis was later disproven for other AOA [29].

Supplementary figures

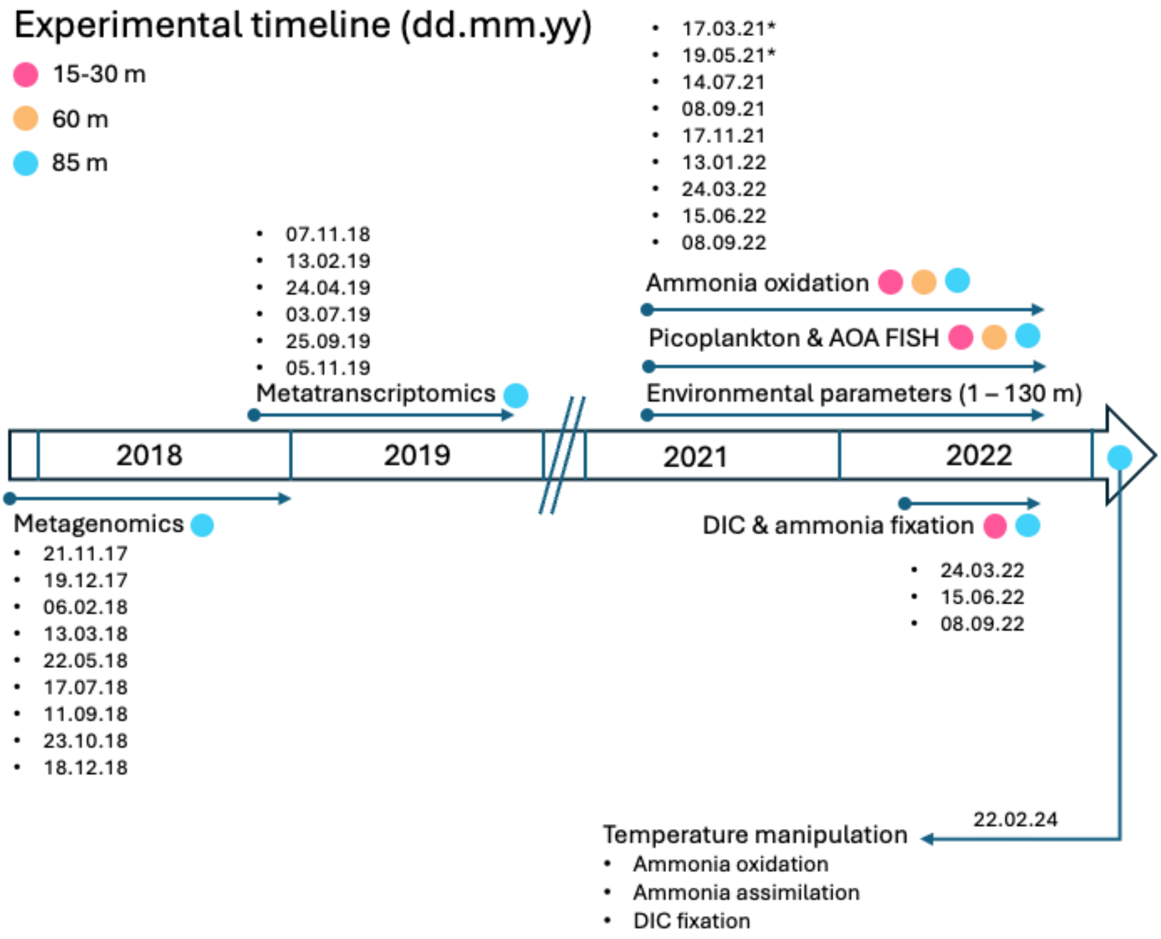

Supplementary Figure 1. Experimental timeline of the individual experimental efforts conducted at the long-term ecological research station of the University of Konstanz (station Wallhausen; 47.75788° N, 9.12617° E) in nortwestern Upper Lake Constance. * - potential ammonia oxidation rates were determined for 85 m only.

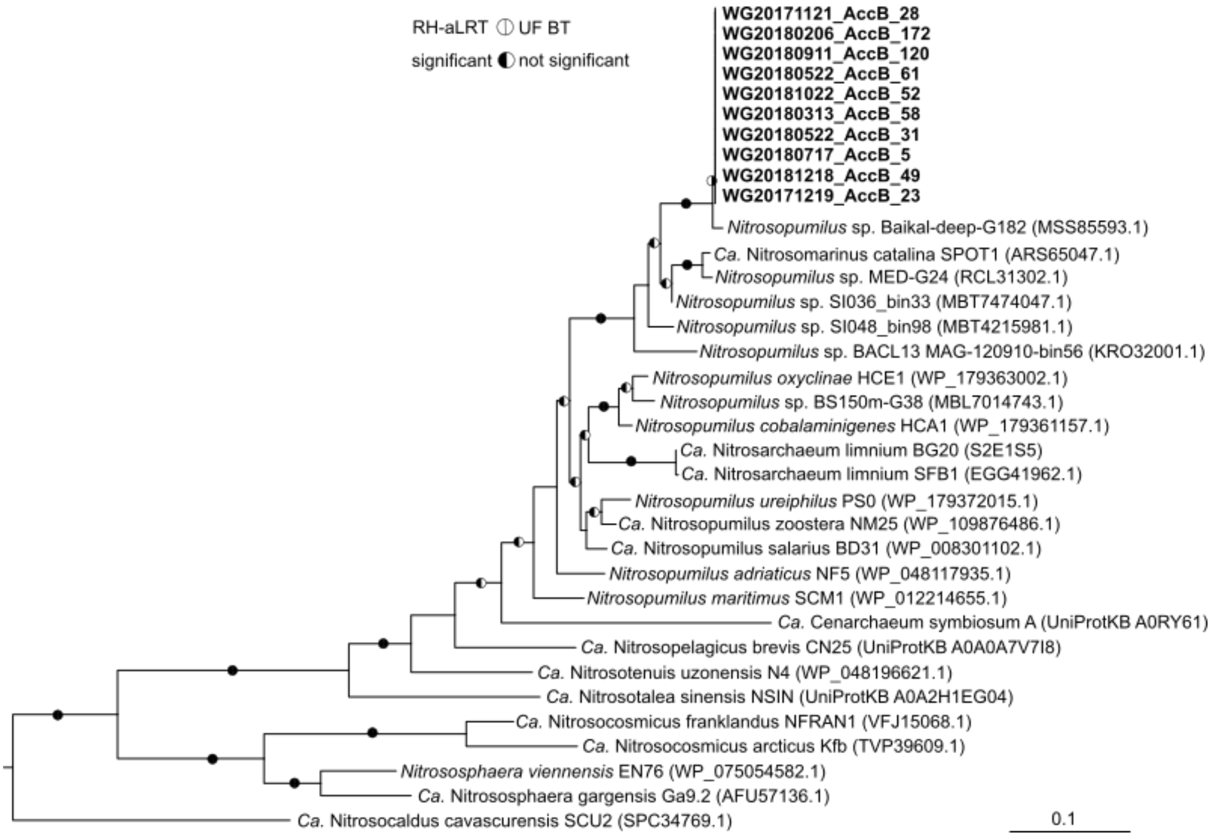

Supplementary Figure 2. Maximum likelihood phylogeny of AccB sequences derived from a gene-centric screening of hypolimnion metagenomes (85 m) of Lake Constance, station Wallhausen. The maximum likelihood tree was constructed using IQ-TREE (Nguyen et al. 2014) as based on 515 unambiguous alignment positions of deduced AccB amino acid sequences. Lake Constance sequences are given in bold, sequences retrieved from public databases are indicated with their respective NCBI accession number. Branch support was tested with the Shimodaira–Hasegawa approximate likelihood-ratio test (SH-aLRT; 1,000 replicates) and ultrafast bootstrap (1,000 replicates) within the IQ-tree software package. Branch support was set as significant at ≥80% for SH-aLRT and ≥95% for ultrafast bootstrap values (black semi-circles for significant and white for non-significant, respectively). AccB of *Haloferax mediterranei*, *Cenarchaeum symbiosum*, and *Thermosulfidibacter takaii* served as outgroup. The scale bar indicates 10% estimated amino acid sequence divergence.

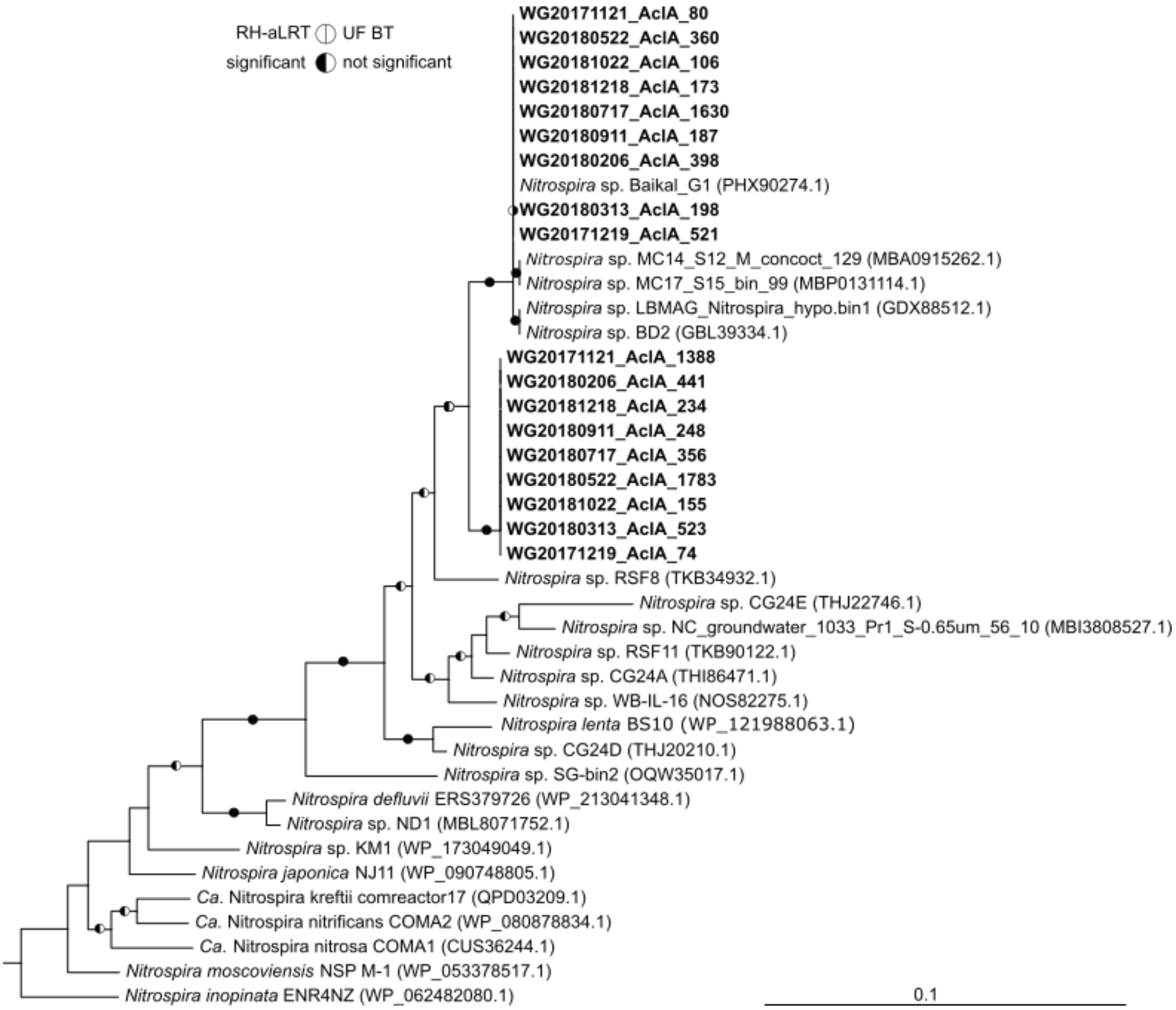

Supplementary Figure 3. Maximum likelihood phylogeny of AclA sequences derived from a gene-centric screening of hypolimnion metagenomes (85 m) of Lake Constance, station Wallhausen. The maximum likelihood tree was constructed using IQ-TREE (Nguyen et al. 2014) as based on 611 unambiguous alignment positions of deduced AclA amino acid sequences. Lake Constance sequences are given in bold, sequences retrieved from public databases are indicated with their respective NCBI accession number. Branch support was tested with the Shimodaira–Hasegawa approximate likelihood-ratio test (SH-aLRT; 1,000 replicates) and ultrafast bootstrap (1,000 replicates) within the IQ-tree software package. Branch support was set as significant at ≥80% for SH-aLRT and ≥95% for ultrafast bootstrap values (black semi-circles for significant and white for non-significant, respectively). AclA of *Chlorobium ferrooxidans* DSM 13031*, Chlorobaculum tepidum* ATCC 49652, *and Chlorobium phaeobacteroides* DSM 266 served as outgroup. The scale bar indicates 10% estimated amino acid sequence divergence.

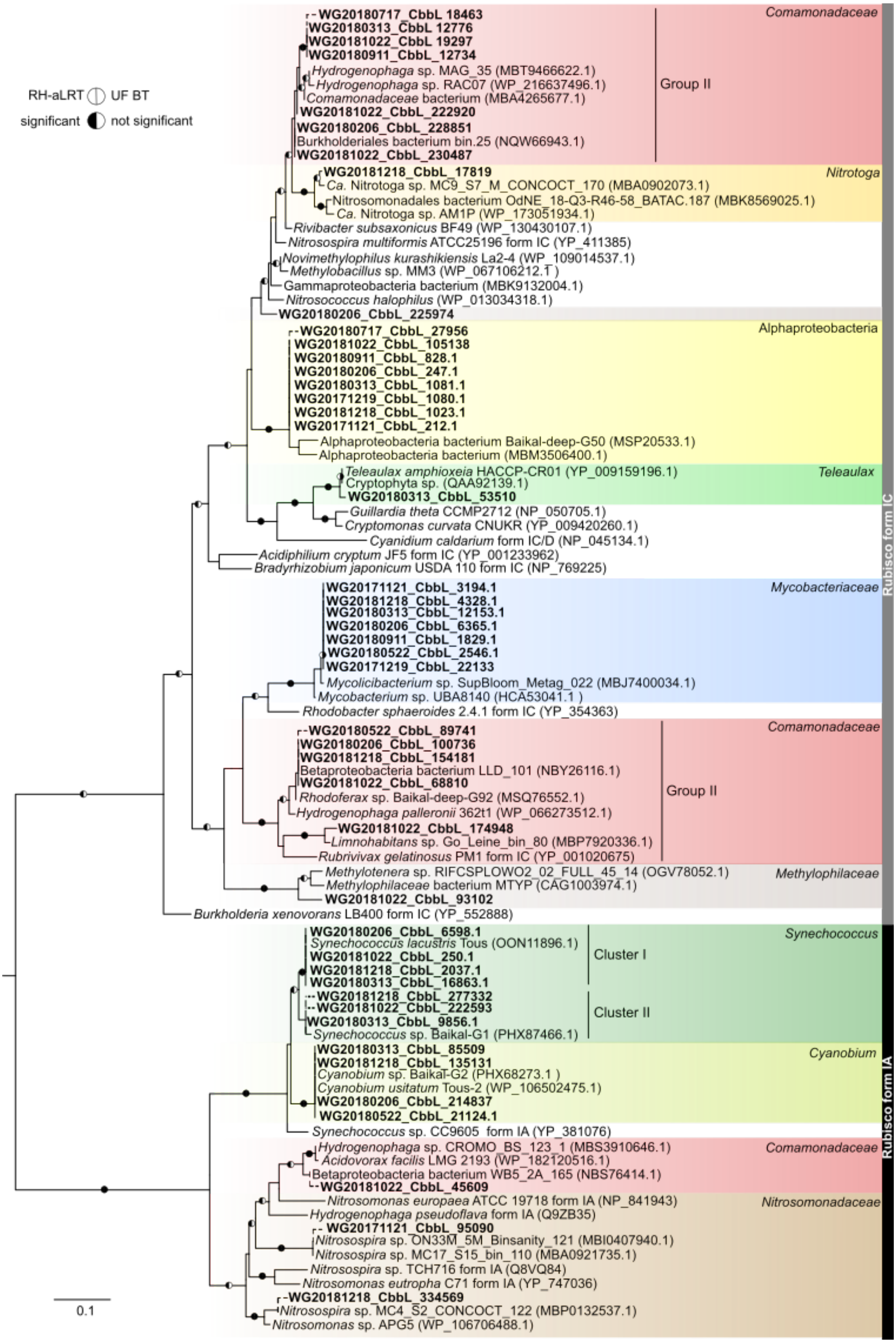
RH-aLRT UF BT
significant not significant
WG20180717_CbbL_18463
WG20180313_CbbL_12776
WG20181022_CbbL_19297
WG20180911_CbbL_12734
Hydrogenophaga sp. MAG_35 (MBT9466622.1)
Hydrogenophaga sp. RAC07 (WP_216637496.1)
Comamonadaceae bacterium (MBA4265677.1)
WG20181022_CbbL_222920
WG20180206_CbbL_228851
Burkholderiales bacterium bin.25 (NQW66943.1)
WG20181022_CbbL_230487
Comamonadaceae
Group II
WG20181218_CbbL_17819
Ca. Nitrotoga sp. MC9_S7_M_CONCOCT_170 (MBA0902073.1)
Nitrosomonadales bacterium OdNE_18-Q3-R46-58_BATAC.187 (MBK8569025.1)
Ca. Nitrotoga sp. AM1P (WP_173051934.1)
Nitrotoga
Rivibacter subsaxonicus BF49 (WP_130430107.1)
Nitrosospira multiformis ATCC25196 form IC (YP_411385)
Novimethylophilus kurashikiensis La2-4 (WP_109014537.1)
Methylobacillus sp. MM3 (WP_067106212.1)
Gammaproteobacteria bacterium (MBK9132004.1)
Nitrosococcus halophilus (WP_013034318.1)
WG20180206_CbbL_225974
WG20180717_CbbL_27956
WG20181022_CbbL_105138
WG20180911_CbbL_828.1
WG20180206_CbbL_247.1
WG20180313_CbbL_1081.1
WG20171219_CbbL_1080.1
WG20181218_CbbL_1023.1
WG20171121_CbbL_212.1
Alphaproteobacteria bacterium Baikal-deep-G50 (MSP20533.1)
Alphaproteobacteria bacterium (MBM3506400.1)
Alphaproteobacteria
Teleaulax amphioxeia HACCP-CR01 (YP_009159196.1)
Cryptophyta sp. (QAA92139.1)
WG20180313_CbbL_53510
Teleaulax
Guillardia theta CCMP2712 (NP_050705.1)
Cryptomonas curvata CNUKR (YP_009420260.1)
Cyanidium caldarium form IC/D (NP_045134.1)
Acidiphilium cryptum JF5 form IC (YP_001233962)
Bradyrhizobium japonicum USDA 110 form IC (NP_769225)
WG20171121_CbbL_3194.1
WG20181218_CbbL_4328.1
WG20180313_CbbL_12153.1
WG20180206_CbbL_6365.1
WG20180911_CbbL_1829.1
WG20180522_CbbL_2546.1
WG20171219_CbbL_22133
Mycolicibacterium sp. SupBloom_Metag_022 (MBJ7400034.1)
Mycobacterium sp. UBA8140 (HCA53041.1)
Mycobacteriaceae
Rhodobacter sphaeroides 2.4.1 form IC (YP_354363)
WG20180522_CbbL_89741
WG20180206_CbbL_100736
WG20181218_CbbL_154181
Betaproteobacteria bacterium LLD_101 (NBY26116.1)
WG20181022_CbbL_68810
Rhodoferax sp. Baikal-deep-G92 (MSQ76552.1)
Hydrogenophaga palleronii 362t1 (WP_066273512.1)
WG20181022_CbbL_174948
Limnohabitans sp. Go_Leine_bin_80 (MBP7920336.1)
Rubrivivax gelatinosus PM1 form IC (YP_001020675)
Comamonadaceae
Group II
Methylotenera sp. RIFCSPLOWO2_02_FULL_45_14 (OGV78052.1)
Methylophilaceae bacterium MTYP (CAG1003974.1)
WG20181022_CbbL_93102
Methylophilaceae
Burkholderia xenovorans LB400 form IC (YP_552888)
Rubisco form IC
WG20180206_CbbL_6598.1
Synechococcus lacustris Tous (OON11896.1)
WG20181022_CbbL_250.1
WG20181218_CbbL_2037.1
WG20180313_CbbL_16863.1
Cluster I
WG20181218_CbbL_277332
WG20181022_CbbL_222593
WG20180313_CbbL_9856.1
Synechococcus sp. Baikal-G1 (PHX87466.1)
Cluster II
Synechococcus
WG20180313_CbbL_85509
WG20181218_CbbL_135131
Cyanobium sp. Baikal-G2 (PHX68273.1)
Cyanobium usitatum Tous-2 (WP_106502475.1)
WG20180206_CbbL_214837
WG20180522_CbbL_21124.1
Cyanobium
Synechococcus sp. CC9605 form IA (YP_381076)
Hydrogenophaga sp. CROMO_BS_123_1 (MBS3910646.1)
Acidovorax facilis LMG 2193 (WP_182120516.1)
Betaproteobacteria bacterium WB5_2A_165 (NBS76414.1)
WG20181022_CbbL_45609
Comamonadaceae
Nitrosomonas europaea ATCC 19718 form IA (NP_841943)
Hydrogenophaga pseudoflava form IA (Q9ZB35)
WG20171121_CbbL_95090
Nitrosospira sp. ON33M_5M_Binsanity_121 (MBI0407940.1)
Nitrosospira sp. MC17_S15_bin_110 (MBA0921735.1)
Nitrosospira sp. TCH716 form IA (Q8VQ84)
Nitrosomonas eutropha C71 form IA (YP_747036)
WG20181218_CbbL_334569
Nitrosospira sp. MC4_S2_CONCOCT_122 (MBP0132537.1)
Nitrosomonas sp. APG5 (WP_106706488.1)
Nitrosomonadaceae
Rubisco form IA
0.1

Supplementary Figure 4. Maximum likelihood phylogeny of CbbL sequences derived from a gene-centric screening of hypolimnion metagenomes (85 m) of Lake Constance, station Wallhausen. The maximum likelihood tree was constructed using IQ-TREE (Nguyen et al. 2014) as based on 274 unambiguous alignment positions of deduced CbbL amino acid sequences. Lake Constance sequences are given in bold, sequences retrieved from public databases are indicated with their respective NCBI accession number. Branch support was tested with the Shimodaira–Hasegawa approximate likelihood-ratio test (SH-aLRT; 1,000 replicates) and ultrafast bootstrap (1,000 replicates) within the IQ-tree software package. Branch support was set as significant at ≥80% for SH-aLRT and ≥95% for ultrafast bootstrap values (black semi-circles for significant and white for non-significant, respectively). RubisCO-like CbbL homologues assembled from the metagenomes served as outgroup. The scale bar indicates 10% estimated amino acid sequence divergence.

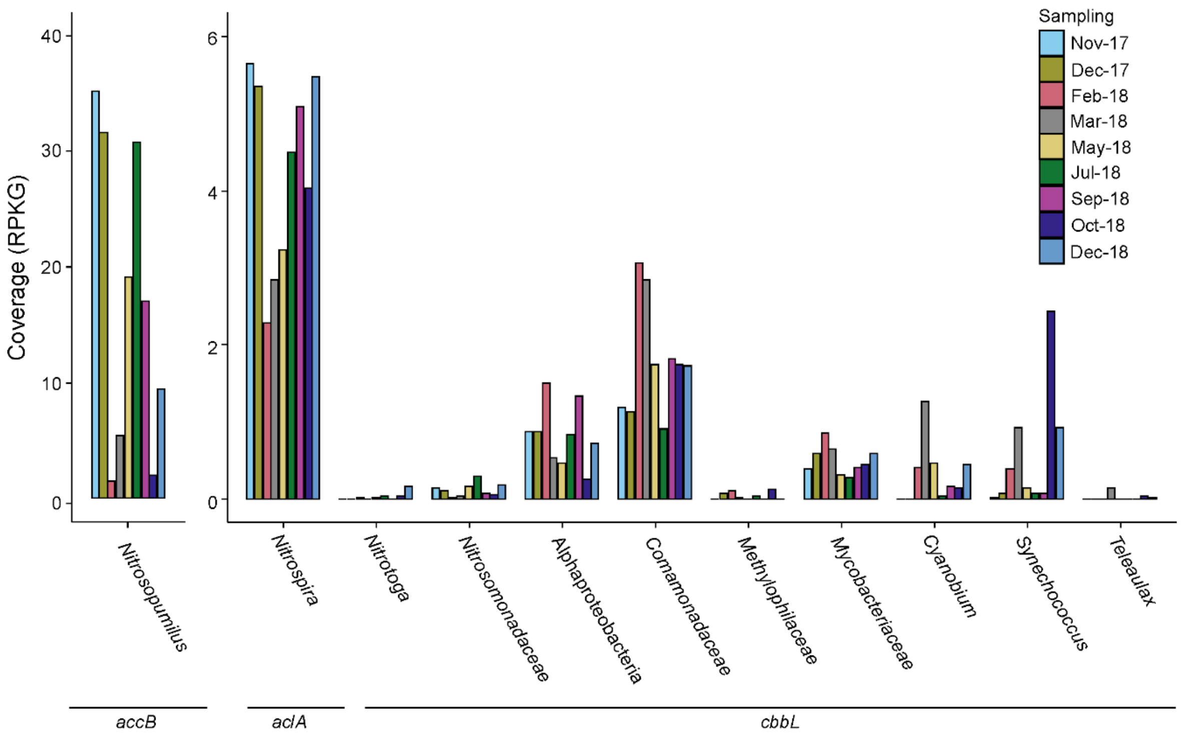

Supplementary Figure 5. Relative abundance of microbial groups encoding DIC fixation pathways in the hypolimnion of Lake Constance across a time series spanning more than one year. Relative abundance was determined from coverage of key marker genes detected in a metagenomic survey at 85 m depth at the station Wallhausen. For the 3HP/4HB cycle, the gene encoding the beta subunit of the acetyl-CoA/propionyl-CoA carboxylase (*accB*), for the rTCA cycle, the gene encoding the alpha-subunit of the ATP-citrate lyase (*aclA*), and for the CBB cycle, the gene encoding the large subunit of ribulose-1,5-bisphosphate carboxylase/oxygenase (RubisCO) (*cbbL*) are shown. Please note the different scale of the y-axes. RPKG = Reads per kilobase of gene per gigabase of metagenome.

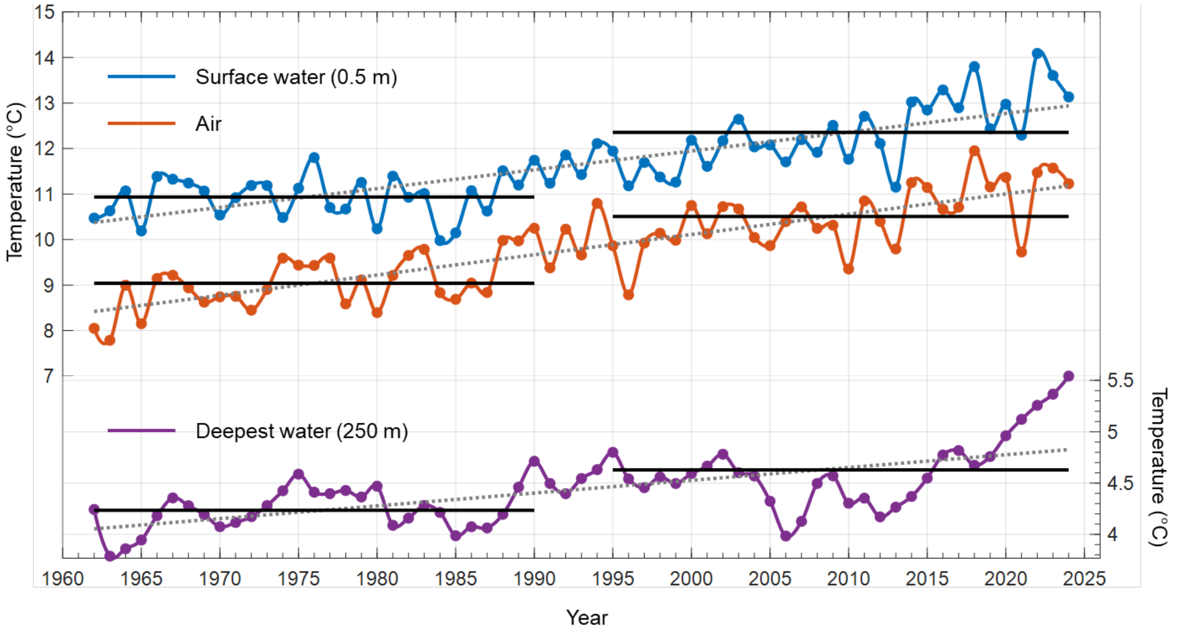

Supplementary Figure 6. Long-term development of annual average temperatures in the air, surface water (0.5 m), and bottom water (250 m) of Lake Constance. Water temperatures recorded at sampling station Fischbach-Uttwil (47.625556° N, 9.384000° E) were taken from the long-term monitoring dataset of the Institute for Lake Research in Langenargen (LUBW - state office for the environment Baden-Württemberg, Germany). Air temperatures were taken from the German Weather Service (www.dwd.de) for the City of Constance. Solid lines represent mean values for the periods of 1962-1990 and 1995-2024. Dashed lines represent linear regressions throughout the respective temperature datasets.

References

1. Musat N, Stryhanyuk H, Bombach P *et al.* The effect of fish and card-fish on the isotopic composition of ^{13}c- and ^{15}n-labeled *pseudomonas putida* cells measured by nanosims. *Syst Appl Microbiol* 2014;**37**:267-76. https://doi.org/10.1016/j.syapm.2014.02.002
2. Meyer NR, Fortney JL, Dekas AE. Nanosims sample preparation decreases isotope enrichment: Magnitude, variability and implications for single-cell rates of microbial activity. *Environ Microbiol* 2021;**23**:81-98. https://doi.org/10.1111/1462-2920.15264
3. Woebken D, Burow LC, Behnam F *et al.* Revisiting n_2 fixation in guerrero negro intertidal microbial mats with a functional single-cell approach. *The ISME Journal* 2014;**9**:485-96. https://doi.org/10.1038/ismej.2014.144
4. Cabello-Yeves PJ, Picazo A, Camacho A *et al.* Ecological and genomic features of two widespread freshwater picocyanobacteria. *Environ Microbiol* 2018;**20**:3757-71. https://doi.org/10.1111/1462-2920.14377
5. Cabello-Yeves PJ, Zemskaya TI, Zakharenko AS *et al.* Microbiome of the deep lake baikal, a unique oxic bathypelagic habitat. *Limnol Oceanogr* 2020;**65**:1471-88. https://doi.org/10.1002/lno.11401
6. Podowski JC, Paver SF, Newton RJ *et al.* Genome streamlining, proteorhodopsin, and organic nitrogen metabolism in freshwater nitrifiers. *mBio* 2022;**13**:e02379-21. https://doi.org/10.1128/mbio.02379-21
7. Rodriguez-R LM, Tsementzi D, Luo C *et al.* Iterative subtractive binning of freshwater chronoseries metagenomes identifies over 400 novel species and their ecologic preferences. *Environ Microbiol* 2020;**22**:3394-412. https://doi.org/10.1111/1462-2920.15112
8. Sabuda MC, Brazelton WJ, Putman LI *et al.* A dynamic microbial sulfur cycle in a serpentinizing continental ophiolite. *Environ Microbiol* 2020;**22**:2329-45. https://doi.org/10.1111/1462-2920.15006
9. Taubert M, Overholt WA, Heinze BM *et al.* Bolstering fitness via co_2 fixation and organic carbon uptake: Mixotrophs in modern groundwater. *The ISME Journal* 2021;**16**:1153-62. https://doi.org/10.1038/s41396-021-01163-x
10. Fixen KR, Starkenburg SR, Hovde BT *et al.* Genome sequences of eight bacterial species found in coculture with the haptophyte *chrysochromulina tobin*. *Genome Announcements* 2016;**4**:e01162-16. https://doi.org/10.1128/genomea.01162-16
11. Schneider D, Zühlke D, Poehlein A *et al.* Metagenome-assembled genome sequences from different wastewater treatment stages in germany. *Microbiology Resource Announcements* 2021;**10**:00504-21. https://doi.org/10.1128/mra.00504-21
12. Anantharaman K, Brown CT, Hug LA *et al.* Thousands of microbial genomes shed light on interconnected biogeochemical processes in an aquifer system. *Nat Commun* 2016;**7**:13219. https://doi.org/10.1038/ncomms13219
13. Cabello-Yeves PJ, Zemskaya TI, Rosselli R *et al.* Genomes of novel microbial lineages assembled from the sub-ice waters of lake baikal. *Appl Environ Microbiol* 2018;**84**:e02132-17. https://doi.org/10.1128/AEM.02132-17
14. Tran PQ, Bachand SC, McIntyre PB *et al.* Depth-discrete metagenomics reveals the roles of microbes in biogeochemical cycling in the tropical freshwater lake tanganyika. *The ISME Journal* 2021;**15**:1971-86. https://doi.org/10.1038/s41396-021-00898-x
15. Sheik CS, Natwora KE, Alexson EE *et al.* *Dolichospermum* blooms in lake superior: DNA-based approach provides insight to the past, present and future of blooms. *J Great Lakes Res* 2022;**48**:1191-205. https://doi.org/10.1016/j.jglr.2022.08.002
16. Parks DH, Chuvochina M, Waite DW *et al.* A standardized bacterial taxonomy based on genome phylogeny substantially revises the tree of life. *Nature Biotechnology* 2018;**36**:996. https://doi.org/10.1038/nbt.4229

17. Altenburger A, Blossom HE, Garcia-Cuetos L *et al.* Dimorphism in cryptophytes—the case of *teleaulax amphioxeia/plagioselmis prolonga* and its ecological implications. *Science Advances* 2020;**6**:eabb1611. https://doi.org/10.1126/sciadv.abb1611

18. Willems A, Busse J, Goor M *et al.* Hydrogenophaga, a new genus of hydrogen-oxidizing bacteria that includes *hydrogenophaga flava* comb. Nov. (formerly *pseudomonas flava*), *hydrogenophaga palleronii* (formerly *pseudomonas palleronii*), *hydrogenophaga pseudoflava* (formerly *pseudomonas pseudoflava* and "pseudomonas carboxy do flava"), and *hydrogenophaga taeniospiralis* (formerly *pseudomonas taeniospiralis*). *Int J Syst Evol Microbiol* 1989;**39**:319-33. https://doi.org/10.1099/00207713-39-3-319

19. Kämpfer P, Schulze R, Jäckel U *et al. Hydrogenophaga defluvii* sp. Nov. And *hydrogenophaga atypica* sp. Nov., isolated from activated sludge. *Int J Syst Evol Microbiol* 2005;**55**:341-44. https://doi.org/10.1099/ijs.0.03041-0

20. Madigan MT, Jung DO, Woese CR *et al. Rhodoferax antarcticus* sp. Nov., a moderately psychrophilic purple nonsulfur bacterium isolated from an antarctic microbial mat. *Arch Microbiol* 2000;**173**:269-77. https://doi.org/10.1007/s002030000140

21. Hahn MW, Kasalický V, Jezbera J *et al. Limnohabitans curvus* gen. Nov., sp. Nov., a planktonic bacterium isolated from a freshwater lake. *Int J Syst Evol Microbiol* 2010;**60**:1358-65. https://doi.org/10.1099/ijs.0.013292-0

22. Šimek K, Kasalický V, Jezbera J *et al.* Broad habitat range of the phylogenetically narrow r-bt065 cluster, representing a core group of the betaproteobacterial genus *limnohabitans*. *Appl Environ Microbiol* 2010;**76**:631-39. https://doi.org/10.1128/AEM.02203-09

23. Kasalický V, Jezbera J, Hahn MW *et al.* The diversity of the *limnohabitans* genus, an important group of freshwater bacterioplankton, by characterization of 35 isolated strains. *PLOS ONE* 2013;**8**:e58209. https://doi.org/10.1371/journal.pone.0058209

24. Zeng Y, Kasalický V, Simek K *et al.* Genome sequences of two freshwater betaproteobacterial isolates,*limnohabitans* species strains rim28 and rim47, indicate their capabilities as both photoautotrophs and ammonia oxidizers. *J Bacteriol* 2012;**194**:6302-03.

25. Kasalický V, Zeng Y, Piwosz K *et al.* Aerobic anoxygenic photosynthesis is commonly present within the genus *limnohabitans*. *Appl Environ Microbiol* 2018;**84**:e02116-17. https://doi.org/10.1128/AEM.02116-17

26. von Kügelgen A, Cassidy CK, van Dorst S *et al.* Membraneless channels sieve cations in ammonia-oxidizing marine archaea. *Nature* 2024;**630**:230-36. https://doi.org/10.1038/s41586-024-07462-5

27. Klotz F, Kitzinger K, Ngugi DK *et al.* Quantification of archaea-driven freshwater nitrification from single cell to ecosystem levels. *The ISME Journal* 2022;**16**:1647-56. https://doi.org/10.1038/s41396-022-01216-9

28. Qin W, Amin SA, Martens-Habbena W *et al.* Marine ammonia-oxidizing archaeal isolates display obligate mixotrophy and wide ecotypic variation. *Proc Natl Acad Sci USA* 2014;**111**:12504-09. https://doi.org/10.1073/pnas.1324115111

29. Kim J-G, Park S-J, Sinninghe Damsté JS *et al.* Hydrogen peroxide detoxification is a key mechanism for growth of ammonia-oxidizing archaea. *Proc Natl Acad Sci USA* 2016 https://doi.org/10.1073/pnas.1605501113
